# Altered ACE2 and interferon landscape in the COVID-19 microenvironment correlate with the anti-PD-1 response in solid tumors

**DOI:** 10.1007/s00018-024-05520-9

**Published:** 2024-12-03

**Authors:** Karthikeyan Subbarayan, Ahmed Al-Samadi, Helene Schäfer, Chiara Massa, Tuula Salo, Katharina Biehl, Christoforos K. Vaxevanis, Kamatchi Ulagappan, Wafa Wahbi, Matthias Reimers, Felix Drexler, Andres Moreira-Soto, Michael Bachmann, Barbara Seliger

**Affiliations:** 1 Medical Faculty, Martin Luther University Halle-Wittenberg, Magdeburger Str. 2, 06112 Halle (Saale), Germany; 2 Institute of Dentistry, School of Medicine, Faculty of Health Sciences, University of Eastern Finland, Joensuu, Finland; 3 Department of Oral and Maxillofacial Diseases, Clinicum, University of Helsinki, Helsinki, Finland; 4 Charité, Institute of Virology, Berlin, Germany; 5 Institute of Radiopharmaceutical Cancer Research, Helmholtz-Zentrum Dresden-Rossendorf, Dresden, Germany; 6 Institute of Translational Immunology, Brandenburg an der Havel, Germany; 7 Cancer and Translational Medicine Research Unit, University of Oulu, Oulu, 90014 Finland; 8 Fraunhofer Institute for Cell Therapy and Immunology, Leipzig, Germany; 9 Institute of Translational Medicine, Medical School Theodor Fontane, Hochstr. 29, 14770 Brandenburg an der Havel, Germany

**Keywords:** SARS-CoV-2, Immune escape, Immune response, Tumors, Immunotherapy

## Abstract

Angiotensensin-converting enzyme-2 (ACE2) is a receptor for SARS-CoV-2, allowing the virus to enter cells. Although tumor patients infected by SARS-CoV-2 often have a worse outcome, the expression, function and clinical relevance of ACE2 in tumors has not yet been thoroughly analyzed. In this study, RNA sequencing (RNA-seq) data from tumors, adjacent tissues and whole blood samples of COVID-19 patients from genome databases and from tumor cell lines and endothelial cells infected with different SARS-CoV-2 variants or transfected with an ACE2 expression vector (ACE2^high^) or mock (ACE2^low^) were analyzed for the expression of ACE2 and immune response relevant molecules in silico or by qPCR, flow cytometry, Western blot and/or RNA-seq. The differential expression profiles in ACE2^high^ vs. ACE2^low^ cells correlated with available SARS-CoV-2 RNA-seq datasets. ACE2^high^ cells demonstrated upregulated mRNA and/or protein levels of HLA class I, programmed death ligand 1 (PD-L1), components of the antigen processing machinery (APM) and the interferon (IFN) signaling pathway compared to ACE2^low^ cells. Co-cultures of ACE2^high^ cells with peripheral blood mononuclear cells increased immune cell migration and infiltration towards ACE2^high^ cells, apoptosis of ACE2^high^ cells, release of innate immunity-related cytokines and altered NK cell-mediated cytotoxicity. Thus, ACE2 expression was associated in different model systems and upon SARS-CoV-2 infection with an altered host immunogenicity, which might influence the efficacy of immune checkpoint inhibitors. These results provide novel insights into the (patho)physiological role of ACE2 on immune response-relevant mechanisms and suggest an alternative strategy to reduce COVID-19 severity in infected tumor patients targeting the ACE2-induced IFN-PD-L1 axis.

**Supplementary Information:**

The online version contains supplementary material available at 10.1007/s00018-024-05520-9.

## Background

Severe acute respiratory syndrome (SARS) coronavirus-2 (SARS-CoV-2) induces the COVID-19 disease first described in China in December 2019, which has caused a global pandemic [1]. SARS-CoV-2 is a single-strand RNA virus belonging to the family of coronaviruses (CoV) and its infection is characterized by fever, pneumonia and respiratory failure with diffuse alveolar damage and mortality rates approximately ten times higher than upon influenza virus infection [2]. The COVID-19 pandemic has caused considerable morbidity and mortality in patients, with increased cancer-related deaths for many tumor types during 2020 and 2021, mainly attributed to COVID-19 as the underlying cause [3, 4].

The SARS-CoV-2 variants influence the treatment and vaccine development due to their distinct effects on immune responses [5]. Since a deregulated immune response may promote virus replication, trigger inflammation and cause immunopathology associated with immune escape [6–9], the interplay between SARS-CoV-2 and the host’s immune system has been postulated to control the disease outcome [5]. This is associated with an altered immune response characterized by inflammatory cytokine production leading to the migration of T cells, monocytes and macrophages to the infection site [10], while NK cells control COVID-19 infection [11] via a TGF-β-dominated immune response [12].

The angiotensin-converting enzyme-2 (ACE2), in combination with the viral spike (S) protein, serves as a gateway for the entry of some coronaviruses, such as HCoV-NL63, SARS-CoV and SARS-CoV-2, into cells [13, 14]. ACE2 is expressed in human tissues, particularly in the epithelium of human lung, oral mucosa and small intestine [15, 16]. In the context of SARS-CoV-2 infection, ACE2 plays a role in the functions of different immune cell subsets [17, 18]. High levels of ACE2 expression are associated with a risk of vulnerability to SARS-CoV-2 infection [19] and with a worse cancer prognosis [20–25]. ACE2 has also been reported as an IFN response gene, leading to an altered interaction between viral infection and host anti-viral responses [26]. Due to their reduced immunity, cancer patients have been suggested as a high-risk group for SARS-CoV-2 infection [27], which was confirmed by a meta-analysis of 38 studies comprising 7094 patients with COVID-19 demonstrating an association of cancer comorbidities with the risk and management of COVID-19 [28, 29].

Increasing evidence exists that viruses influence tumor growth by modulating different signal cascades leading to decreased apoptosis, immune suppression and increased angiogenesis. The cross-talk between viral proteins and inflammatory mediators results in an altered TME associated with tumor progression [27, 30–33]. Bioinformatics analyses of different cancers demonstrated a positive correlation between elevated ACE2 expression levels, immune cell infiltration and patients’ prognosis [25]. Severe or critical COVID-19 is linked to increased serum levels of pro-inflammatory cytokines and altered composition of immune cell subpopulations [34]. This aggressive inflammatory response and cytokine storm contribute to severe systemic tissue damage and mortality. Blocking the cytokine-mediated inflammatory cell death may benefit patients with COVID-19 or other infectious diseases by limiting tissue damage [35]. During the SARS-CoV-2 infection of cancer patients, distinct immune mechanisms were identified which impact the selection and success of immunological-based therapies, such as immune checkpoint inhibitors (ICPi) and the patients’ outcome [36]. It is hypothesized that the high expression of ACE2 in tumors may affect the immune response and the ICPi efficacy in COVID-19 patients. Therefore, it is crucial to gain a better understanding of how ACE2 affects immune responses in cancer patients to identify those at high-risk and develop immunotherapeutic approaches to enhance CD8^+^ T cell responses.

In vitro models of ACE2-transfected tumor cells and bioinformatics analyses of public datasets suggest that there is a link between ACE2 and PD-L1 overexpression and inhibitors of the PD1/PD-L1 axis in humans infected with SARS-CoV-2 might balance host restriction, tissue tolerance, viral enhancement mechanisms as well as improve immune cell infiltration into tumors.

## Materials and methods

### Cell culture and transfection

The human tumor cell lines MCF-7 (breast cancer (BC)), A549 (lung cancer), RKO (colorectal cancer (CRC)) and the endothelial cell line EA.Hy926 were purchased from the American Type Culture Collection (ATCC, Manassas, USA). All cell lines were cultured in RPMI1640 medium supplemented with 1% 100 mM glutamine, 10% fetal calf serum (FCS, PAN-Biotech, Aidenbach, Germany) and respective antibiotics.

The different human tumor epithelial and endothelial cell lines were transfected with an ACE2 expression vector (ACE2^high^) (Addgene, Watertown, USA) using Effectene Transfection Reagent (Qiagen, Hilden, Germany) according to the manufacturer’s instructions, while transfection with a mock vector (ACE2^low^) served as a control. Stable ACE2 transfectants (ACE2^high^) and vector controls (ACE2^low^) were maintained in complete culture medium supplementation with G418 (PAA Laboratories GmbH, Cölbe, Germany).

### qPCR analysis

Total cellular RNA from 1 to 5 × 10^6^ tumor cells was isolated and reverse transcribed into cDNA as recently described [37]. qPCR was performed on a Rotor-Gene 6000 system (Qiagen, Hilden, Germany) employing the platinum SYBRGreen qPCR Supermix UDG (Thermo Fisher, Waltham, USA) using a standard protocol. The sequence of primers for HLA class I components, IFN signaling molecules, PD-L1 and ACE2-regulated genes and the conditions used are listed in Supplementary Table 1. Data were analyzed using a comparative quantification mode of the Bio-Rad CFX Maestro Software 2.3. qPCR analyses were performed with RNA from at least three independent experiments.

### Western blot analysis

For Western blot analysis, 30 µg protein/sample was separated by SDS-PAGE, transferred to nitrocellulose membranes (Schleicher & Schuell, Dassel, Germany) followed by staining with the antibodies (Abs) directed against ACE2, TAP1 and IRF1 as recently described [38]. Equal protein loading was determined by staining the blot with an anti-GAPDH monoclonal antibody (mAb) (Cell Signaling Technology, Danvers, USA). A horse reddish peroxidase (HRP)-conjugated secondary antibody was used before the visualization of proteins by chemiluminescence using an ECL-based system.

### Flow cytometry

For flow cytometry, tumor cells were stained with fluorescence-labeled anti-human pan-HLA class I and PD-L1 mAbs or the appropriate isotype control for 30 min. After washing twice with buffer, HLA class I and PD-L1 expression (Invitrogen, Waltham, USA) were determined on a NAVIOS flow cytometer (Beckman Coulter, Brea, USA). Data were analyzed using the Kaluza Software and expressed as mean specific fluorescence intensity (MFI).

### mRNA sequencing and data analyses

Sample preparation and bioinformatics were performed according to the procedures described in previous studies [39]. Reference genome and gene model annotation files were directly downloaded from the genome website (NCBI/UCSC/Ensembl). Gene expression levels were quantified using HTSeq v0.6.1 and FPKM of each gene was calculated based on the length of the gene and read counts mapped to this gene [40].

Initial analysis of differential gene expression (DGE) between ACE2^low^ and ACE2^high^ MCF7 cells was performed by Novogene using the DESeq2 R package (2_1.6.3). DESeq2 provides statistical routines for determining DEG in digital gene expression data using a model based on the negative binomial distribution. The resulting p-values were adjusted using Benjamini and Hochberg’s approach for controlling the False Discovery Rate (FDR). Genes with an adjusted p-value (*P*_adj_) < 0.05 found by DESeq2 were assigned as differentially expressed. Gene Ontology (GO) enrichment analysis of differentially expressed genes was implemented by the overrepresentation analysis function in the clusterProfiler R package, in which gene length bias was corrected. GO terms with corrected *P*_adj_ value < 0.05 were considered significantly enriched.

### Microfluidic chip assay

The ACE2^high^ and ACE-2^low^ MCF-7 cells were stained with CellTrace™ Far Red (Invitrogen, Thermo Fisher) according to the manufacturer’s instructions for the microfluidic chip assay. The cells were then suspended in human-tumor based matrix myogel/fibrin gel using 2.4 mg/ml myogel (lab made), 0.5 mg/ml fibrinogen (Merck, Darmstadt, Germany), 33.3 µg/ml aprotinin (Sigma-Aldrich) and 0.3 U/ml thrombin (Sigma-Aldrich) diluted in total RPMI1640. 5 µM of IncuCyte caspase-3/7 green (Sartorius, Göttingen, Germany) was added to detect apoptotic cells. The ACE2^high^ and ACE-2^low^ MCF-7 cells were divided into a control group without drug and an ICPi-treated group (0.5 µM nivolumab). 2 µL of each cell suspension containing 500 cells in the gel were loaded into separate small “cancer cell channels” of the microfluidic chip as described before [41].

Peripheral blood mononuclear cells (PBMNCs) were isolated from blood buffy coats of healthy donors provided by the Finnish Red Cross by gradient density centrifugation. PBMNCs were stained with CellTrace™ Violet (Invitrogen) according to the manufacturer’s instructions. Cell viability and number were determined by trypan blue staining utilizing CellCountess (Invitrogen). After staining, cells were suspended in the cell culture media supplied with 10 ng/ml recombinant human IL-2 (BioLegend, San Diego, California, USA) and 5 µM caspase-3/7 green (Sartorius). PBMNCs were divided into the following groups: control without drug, and 0.5 µM nivolumab. 100 µL of cell suspension containing 100.000 viable PBMNCs was added to the larger ‘PBMNCs channels’ of the chip as recently described [41]. In controls without PBMNCs, 100 µL of cell culture media containing 5 µM caspase-3/7 green was injected.

After injections, the chips were incubated for 72 h in a cell culture laminar and imaged daily using Nikon Ti-E with Alveole Primo microscope (Nikon, Tokyo, Japan) connected to Hamamatsu Orca Flash 4.0 LT B&W camera (Hamamatsu Photonics, Hamamatsu, Japan). The conditioned media was then collected from the chips and stored at -80 °C until further analyses.

### Cytokine release

Conditioned media from the microfluidic chips were collected for cytokine profiling using Abcam FirePlex Service (Boston, USA). Analysis was performed utilizing FirePlex^®^-96 Key Cytokines (Human) Immunoassay Panel (Abcam, Cambridge, UK), which detects the following 17 cytokines: granulocyte-macrophage colony-stimulating factor (CSF2, GM-CSF), interleukin-(IL)1B, 2, 4, 5, 6, 9, 10, 12 A, 13 and 17 A, CXCL8, IFNG, monocyte chemoattractant protein-1 (MCP-1, CCL2), macrophage inflammatory protein 1 alpha (MIP1-α, CCL3), macrophage inflammatory protein 1 beta (MIP1-β, CCL4) and tumor necrosis factor (TNF)-alpha. Each sample was analyzed in duplicate.

### NK cell assays and co-cultivation

Human PBMNCs were stimulated for 18 h with 1 ng/ml IL-12, 5 ng/ml IL-15 (both from Immunotools, Friesoythe, Germany) and 50 ng/ml IL-18 (Biovision, Milpitas, CA, USA) in X–vivo15 (Lonza) medium followed by their incubation with target cells for a CD107a degranulation assay. The anti-CD107a Ab was added after 1 h of co-culture, followed by staining of cells after 4 h with mAbs directed against CD3, CD16 and CD56 (BioLegend) to identify NK cells and determine total NK cell activity.

### *Datasets and* in silico *analysis*

Whole blood transcriptomic data from 24 healthy controls and 62 COVID-19 patients [42, 43] were analyzed using COVID19db (ID: COVID000010). The baseline characteristics for patients, such as age, gender and blood parameters, were previously described [42]. Metadata from a cohort of SARS-CoV-2 other respiratory viruses, such as human parainfluenza virus 3 (HPIV3), respiratory syncytial virus (RSV) and mutant influenza A virus (IAVdNS) infected cells, as well as COVID-19 positive lung biopsies (GEO accession: GSE147507) were analyzed by using ImmGen of Immunological Genome Project [26, 44, 45]. Single-cell RNA-seq data of patients with severe COVID-19 peripheral blood were retrieved from ImmGen [46] and CZ CELLxGENE Discover. Metadata from a cohort of BC (1097 samples) and Pan-cancer (11003 samples) were analyzed from The Cancer Genome Atlas (TCGA) (portal: https://portal.gdc.cancer.gov) [47] using R2: Genomics analysis and visualization platform (http://r2.amc.nl). The gene expression patterns of ACE2, HLA class I APM, IFN pathway components and PD-L1 were retrieved from the above datasets.

### Statistical analysis

Microsoft Excel-Office 365, BioRender and R (RStudio 3.0) were used for graphical representations, Student’s t-test and one-way ANOVA. Flow cytometer output for cytokine release was analyzed using FirePlex™ Analysis Workbench software (https://www.abcam.com/kits/fireplex-analysis-workbench-software). A p-value of < 0.05 was considered as significant (*, *p* < 0.05; **, *p* < 0.01; ***, *p* < 0.001).

## Results

### Increased expression levels of ACE2 after SARS-CoV-2 infection

**Fig. 1:**
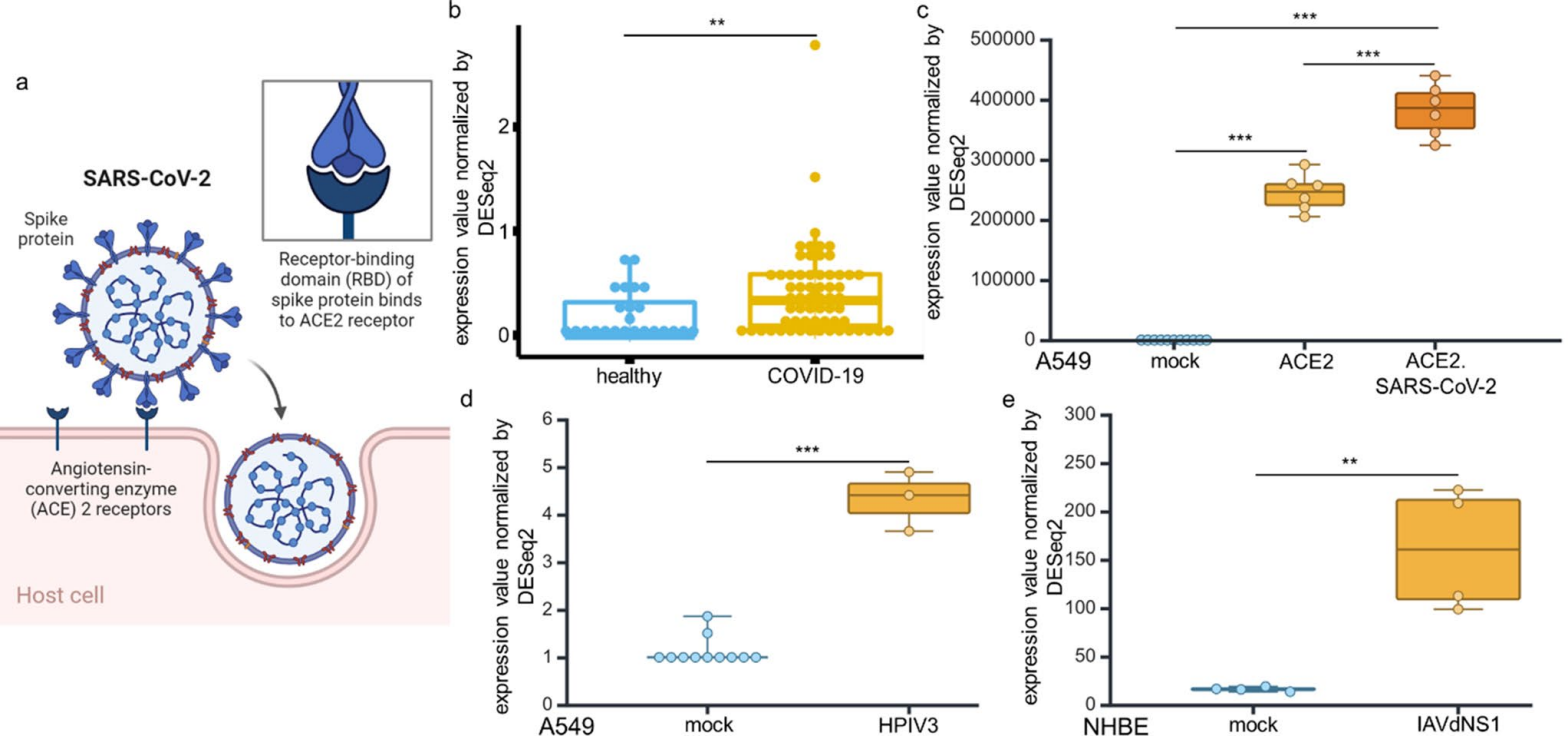
High levels of ACE2 expression in blood samples from COVID-19 patients and SARS-CoV-2 infected cell lines compared to their uninfected counterparts. **a** ACE2 is an entry receptor for SARS-CoV-2 and a key molecule for understanding the pathophysiology of COVID-19. **b** ACE2 expression in blood samples from 24 healthy controls and 62 COVID-19 patients. The data were obtained from whole blood transcriptomic data (COVID19db). **c**, **d** ACE2 expression in A549 lung carcinoma epithelial cells transfected with an ACE2 expression vector or infected with SARS-CoV-2 (C) and infected with HPIV3 (D) (GEO: GSE147507). **e** Expression of ACE2 in normal human bronchial epithelial (NHBE) cells infected with IAVdNS1 (GEO: GSE147507). t.test, *p* > 0.05; *: p < = 0.05; **: p < = 0.01; ***: p < = 0.001; ****: p < = 0.0001

ACE2, which is the molecular pathway through which SARS-CoV-2 enters host cells (Fig. 1a), showed significantly higher expression (Log2FC 0.22; p value 0.01) in blood samples of COVID-19 patients. This was determined by bioinformatics analyses of transcriptomic data obtained from 24 healthy controls and 62 COVID-19 patients (COVID19db ID: COVID000010) (Fig. 1b). The increased levels of ACE2 mRNA in blood samples from COVID-19 patients were similar to those observed in A549 lung carcinoma epithelial cells following ACE2 transfection and/or SARS-CoV-2 infection (Fig. 1c; GEO: GSE147507). Furthermore, increased ACE2 expression levels were also found in response to infections with other respiratory viruses (GEO: GSE147507), such as HPIV3 infection of A549 cells (Fig. 1d) and IAVdNS1 infection of normal human bronchial epithelial (NHBE) cells (Fig. 1e).

### Identification and function of ACE2-regulated genes in MCF-7 BC cells

**Fig. 2:**
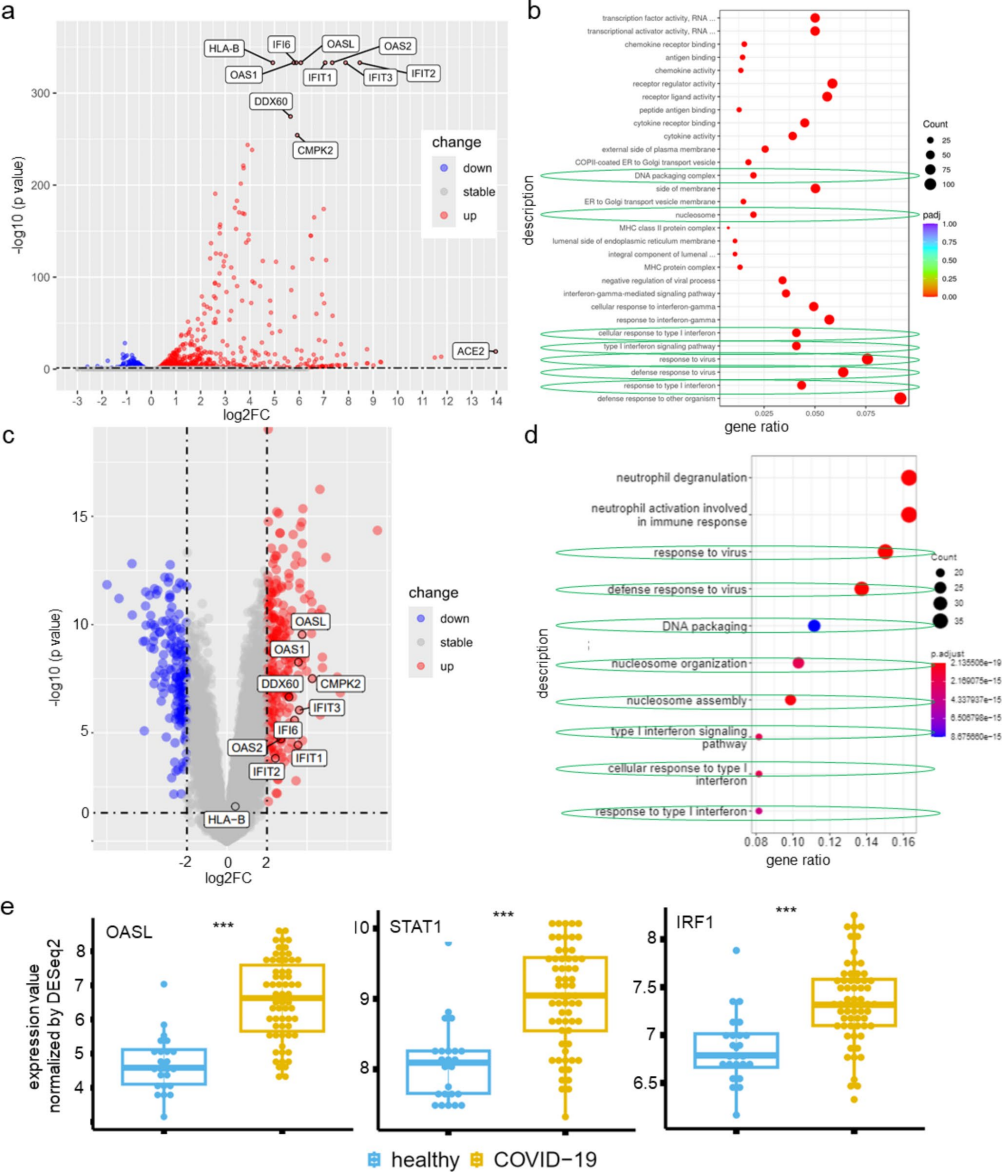
Comparison of transcriptional profiles in ACE2^high^ vs. ACE2^low^ MCF-7 cells and COVID-19 PBMNCs using RNA Sequencing. **a** Volcano plot of the DEGs between ACE2^high^ and ACE2^low^ MCF-7 groups. Significantly down-regulated genes are marked in dark blue, significantly upregulated genes in red and non-significantly regulated genes in grey. **b** The top 20 enriched GO terms from upregulated genes of ACE2^high^ vs. ACE2^low^ MCF-7 cells. **c** Volcano plot of the DEGs between COVID-19 and healthy blood samples (COVID19db). **d** The top 10 enriched GO terms from upregulated genes between COVID-19 and healthy blood samples. The enriched GO terms commonly found in both ACE2^high^ and COVID-19 were represented by green circles. **e** mRNA expression profiles of commonly upregulated genes in the top five GOs enrichments of ACE2^high^ blood samples (COVID19db) from COVID-19 patients. The gene expressions of OASL, STAT1, and IRF1 are shown here as representative

RNA-seq analysis of ACE2^high^ and ACE2^low^ MCF-7 cells revealed a total of 2801 differentially expressed genes (DEGs) (padj < 0.05) with 1445 significantly upregulated and 1356 significantly downregulated genes (Fig. 2a). Gene ontology (GO) enrichment analysis was performed using the DEGs to assess the functional categories of biological process (BP), molecular function (MF) and cellular component (CC). The top 20 significantly enriched GO terms of the upregulated genes in ACE2^high^ and ACE2^low^ MCF-7 cells include the categories defense response to other organism’ (ontology: BP; gene ratio 108/1174; p-value 2.57E-37), ‘cytokine activity’ (ontology: MF; gene ratio 46/1178; p-value 1.84E-16) and ‘MHC protein complex’ (ontology: CC; gene ratio 16/1214; p-value 5.09E-16) (Fig. 2b). The top 20 enriched GO terms of downregulated genes in ACE2^low^ MCF-7 cells contain ‘sister chromatid segregation’ (ontology: BP; gene ratio 60/1176; p-value 7.62E-16), ‘structural constituent of ribosome’ (ontology: MF; gene ratio 41/1177; p-value 1.37E-11) and ‘chromosomal region’ (ontology: CC; gene ratio 80/1236; p-value 2.95E-19) (Supplementary Fig. 1). The top 10 upregulated genes by ACE2 were IFI6, IFIT1, IFIT2, IFIT3, OAS2, OASL, HLA-B, OAS1, DDX60 and CMPK2, the top 10 downregulated genes were SCD, ABCG1, SREBF1, FGFR4, PHGDH, FBXO27, PREX1, CRAT, AIF1L and PXMP4. Disease annotation of the top upregulated genes demonstrated a link to viral infections (Supplementary Table 2), most commonly to influenza (disease id: C0021400) (Supplementary Fig. 2A), while the downregulated genes were annotated to BC (malignant tumor of breast (disease id: C0006142) and breast carcinoma (disease id: C0678222) (Supplementary Fig. 2B). The ACE2-mediated differential expression profiles were independently confirmed for selected DEGs ACE^high^ and ACE^low^ cell systems by qPCR using DEG-specific primers (data not shown).

### Correlation of the GO terms and DEGs between ACE2high MCF-7 cells and SARS-CoV-2-infected PBMNCs

Comparison of the GO terms of the significantly upregulated genes in ACE2^high^ vs. ACE2^low^ MCF-7 cells with those in whole blood obtained from 62 COVID-19 patients and 24 healthy volunteers (COVID19db ID: COVID000010) demonstrated that 9/10 selected upregulated genes in ACE2^high^ MCF-7 cells were expressed at higher levels in blood samples of COVID-19 patients as visualized by a volcano plot (Fig. 2c). Additionally, 8/10 GO terms were commonly enriched in both ACE2^high^ MCF-7 cells and blood samples of COVID-19 patients, as indicated by green circles. These enriched GO terms included ‘response to virus’, ‘nucleosome’, and ‘type I interferon signaling pathway’ (Fig. 2d). As expected, the GO terms ‘neutrophil degranulation and neutrophil activation’ were only found in blood samples of COVID-19 patients, but not in ACE2^high^ MCF-7 cells. The analysis further focused on the significantly upregulated genes within the top five GO terms, namely ‘defense response to other organism’, ‘response to type I IFN’, ‘defense response to virus’, ‘response to virus’, and ‘type I IFN signaling pathway’. Notably, 14 common genes upregulated in ACE2^high^ MCF-7 cells within these top five GO enrichments (Supplementary Fig. 3) were also enhanced in blood samples from COVID-19 patients (COVID19db). These genes include OAS1 (Log2FC 1.78; p value 5.47E-09), OAS2 (Log2FC 1.34; p value 0), OAS3 (Log2FC 1.67; p value 0), OASL (Log2FC 1.87; p value 2.91E-10), STAT1 (Log2FC 0.93; p value 9.34E-08), IFITM3 (Log2FC 1.61; p value 5.94E-09), IRF1 (Log2FC 0.48; p value 3.38E-06), IRF2 (Log2FC 0.19; p value 0.01), IRF7 (Log2FC 1.32; p value 6.86E-07), IRF9 (Log2FC 0.54; p value 0.001), BST2 (Log2FC 0.68; p value 0), IFITM1 (Log2FC 1.19; p value 2.26E-08), IFITM2 (Log2FC 0.67; p value 4.91E-06) and NLRC5 (Log2FC 0.36; p value 0.002). All 14 genes upregulated in genes of ACE2^high^ MCF-7 cells were statistically higher (*p* < 0.05) in COVID-19 blood samples compared to that of healthy controls (Fig. 2e). Additionally, the top 10 up- and downregulated genes of ACE^high^ MCF-7 cells were compared to those of SARS-CoV-2-infected Calu3, A549 and NHBE cells (Supplementary Fig. 4A) as well as to infection with other respiratory viruses, such as IAV, IAVdNS1, HPIV3, and RSV (Supplementary Fig. 4B). Interestingly, except for IFIT2 in SARS-CoV-2-infected NHBE cells, the expression of the top 10 genes in ACE2^high^ MCF-7 cells exhibited a similar increased trend upon viral infections. Among the top 10 downregulated genes, SREBF1, FGFR4, CRAT and PXMP4 showed a similar decrease following different viral infections. Hence, the global transcriptomic profile and functional annotations of ACE2^high^ MCF-7 cells were mainly comparable to those of SARS-CoV-2-infected cells and COVID-19 patients.

### Upregulation of HLA class I surface expression after ACE2 overexpression and SARS-CoV-2 infection

**Fig. 3:**
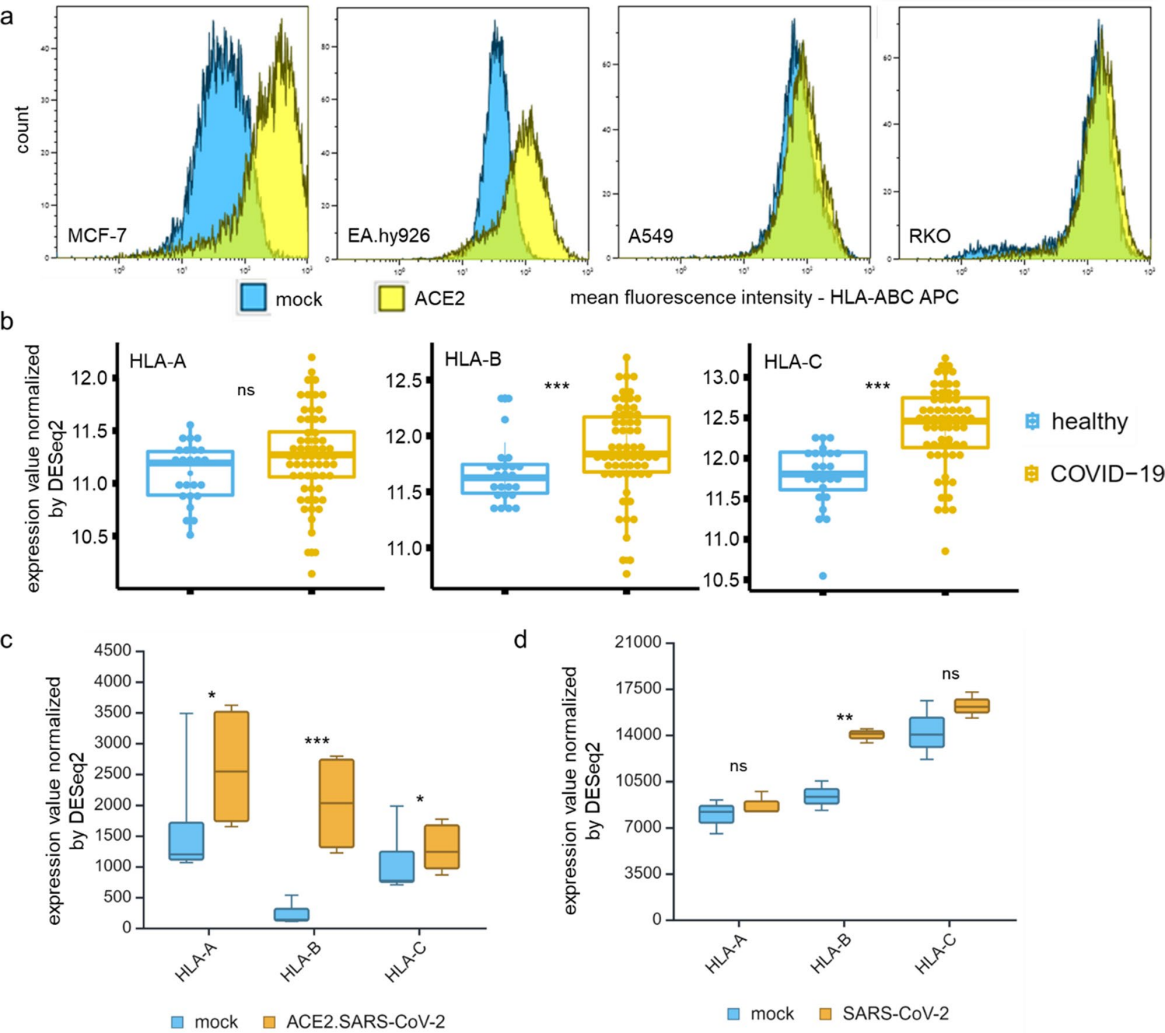
Impact of ACE2 overexpression and respiratory viral infections on the expression of HLA class I. **a** The ACE2 transfectants and mock controls of EA.Hy926, A549, RKO and MCF-7 cells were analyzed for HLA-I surface expression by flow cytometry described in Material and Methods. The results are presented as a histogram and MFI of HLA-ABC (*n* = 3). **b** HLA-A, -B, and -C mRNA expression in blood samples of 62 COVID-19 patients vs. 24 healthy controls (COVID19db). **c**, **d** Increased HLA-A, -B and – C expression levels in SARS-CoV-2-infected cells (GEO accession: GSE147507), ACE2^high^ A549 cells (c) and Calu3 (d)

Since the impact of SARS-CoV-2 infection relevant molecules on the expression of immunemodulatory molecules has thoroughly not been analyzed, we first determined the effect of ACE2 overexpression in MCF-7, RKO, A549 and EA.Hy926 cells on the expression of HLA class I surface antigens using flow cytometry. As shown in Fig. 3a, ACE2 overexpression resulted in an increased HLA class I surface expression in all cell lines, but the levels highly varied. Notably, MCF-7 and EA.Hy926 cells exhibited higher HLA class I expression compared to A549 and RKO cells, which might be due to differences in the cancer types or other factors involved in the regulation of HLA class I antigens. This finding is consistent with high levels of HLA-B (Log2FC 0.2; p value 0.02) and -C (Log2FC 0.59; p value 1.17E-06) expression in blood samples of COVID-19 patients compared to healthy controls (Fig. 3b).

These data were confirmed by RNA-seq results obtained from lung biopsies of COVID-19 patients (GEO: GSE1488290), which displayed a similar correlation with higher mRNA levels of HLA class I antigens (Supplementary Fig. 5). SARS-CoV-2 infection of ACE2^high^ A549 cells (Fig. 3c) and infection with other respiratory viruses, such as IAV, IAVdNS1, HPIV3 and RSV, upregulated HLA class I antigens (Supplementary Fig. 6) when compared to the uninfected controls. HLA-B, but not HLA-A and HLA-C antigens were enhanced in SARS-CoV-2-infected Calu3 cells (Fig. 3d).

### Association of the ACE2-mediated upregulation of HLA class I surface antigens with increased APM and IFN signaling component expression

**Fig. 4:**
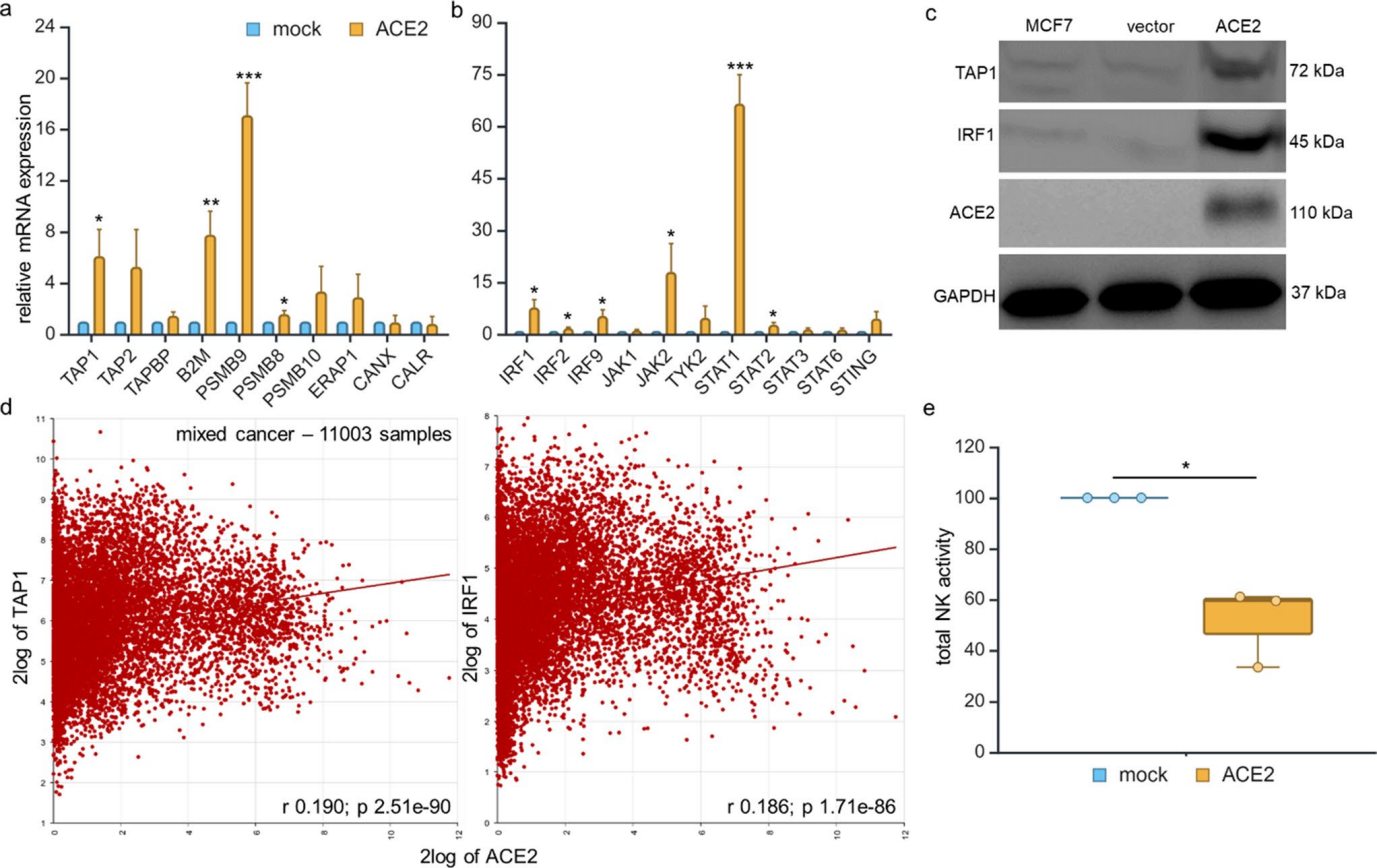
Increased APM and IFN signaling components in ACE2 transfectants and their impact on NK cell activity. **a** The ACE2 transfectants were analyzed for the expression of major HLA-I APM components using qPCR as described in Material and Methods. The results are presented as x-fold upregulation of APM components in ACE2 transfectants vs. mock controls (set = 1). **b** The ACE2 transfectants were analyzed by qPCR for the expression of type I and type II IFN signaling components. The results are represented as an x-fold induction of the expression of IFN signaling components in ACE2 transfectants compared to mock controls (set = 1). **c** A representative Western blot analysis of ACE^high^, mock transfected and potential cells using anti-TAP1 as loading control anti-IRF1 and ACE2 antibodies is shown, staining with an anti-GAPDH antibody served as loading control. **d** In silico analysis of TCGA data compared ACE2 expression to the expression of the APM component, TAP1 and IFN component, IRF1 in a pan-cancer dataset (11003 samples). **e** Reduced NK cell activity in ACE2^high^ vs. ACE2^low^ MCF-7 cells. CD107a degranulation assay was performed by co-culture with NK cells from three different donors with ACE2^low^ vs. ACE2^high^ MCF-7 cells as described in Material and Methods. The mean ± SE of the CD107a degranulation of ACE2^low^ vs. ACE2^high^ MCF-7 cells using NK cells representing total NK cell activity are shown

In order to determine whether the ACE2-mediated increase of HLA class I surface expression was due to an enhanced expression of HLA class I APM components, the human ACE2^high^ and ACE2^low^ model systems were analyzed for the mRNA and protein expression of the major HLA class I APM molecules, such as the transporter associated with antigen processing (TAP)1, TAP2, TAPBP, β_2_-microglobulin (B2M), the IFN-γ inducible proteasome subunits, the low molecular weight proteins PSMB8, PSMB9 and PSMB10 as well as the chaperones calreticulin (CALR) and calnexin (CANX). With the exception of calnexin, calreticulin and tapasin, an ACE2-mediated upregulation of the mRNA expression of all other HLA class I APM components analyzed was detected (Fig. 4a).

Despite SARS-CoV-2 infection has been reported to influence cytokine signaling, including the IFN signaling pathway [48], and IFN-γ has been shown to increase ACE2 surface expression [49], a possible link between ACE2 overexpression and IFN signaling in tumors has not yet been determined. Expression analyses of various IFN type I and II signaling components revealed a strong upregulation of the mRNA expression of IRF1, IRF9, JAK2, STAT1, STING and TYK2 in ACE2^high^ transfectants compared to ACE2^low^ mock controls (Fig. 4b). These data were confirmed by Western blot analyses as representatively shown for TAP1 and IRF1 with an increased TAP1 and IRF1 protein expression in ACE2^high^ vs. ACE2^low^ cells (Fig. 4c).

The possible link between ACE2 expression and immune response relevant profiles was also examined by in silico analyses of cancer genome databases. As shown in Fig. 4d and Supplementary Table 3, a positive correlation of ACE2 with the expression of components of the HLA class I APM as well as the IFN type I and II pathways in pan-cancer and breast cancer samples was found.

The functional relevance of the upregulation of HLA class I by ACE2 was determined by co-culture of ACE2^low^ and ACE2^high^ MCF-7 cells by a CD107a degranulation assay and the NK cell-mediated recognition was determined. As expected, a decreased cytotoxicity of NK cells of ACE2^high^ MCF-7 cells compared to ACE2^low^ MCF-7 cells was detected (Fig. 4e).

### Correlation of ACE2 expression with the expression of the immune checkpoint molecule PD-L1 (CD274)

**Fig. 5:**
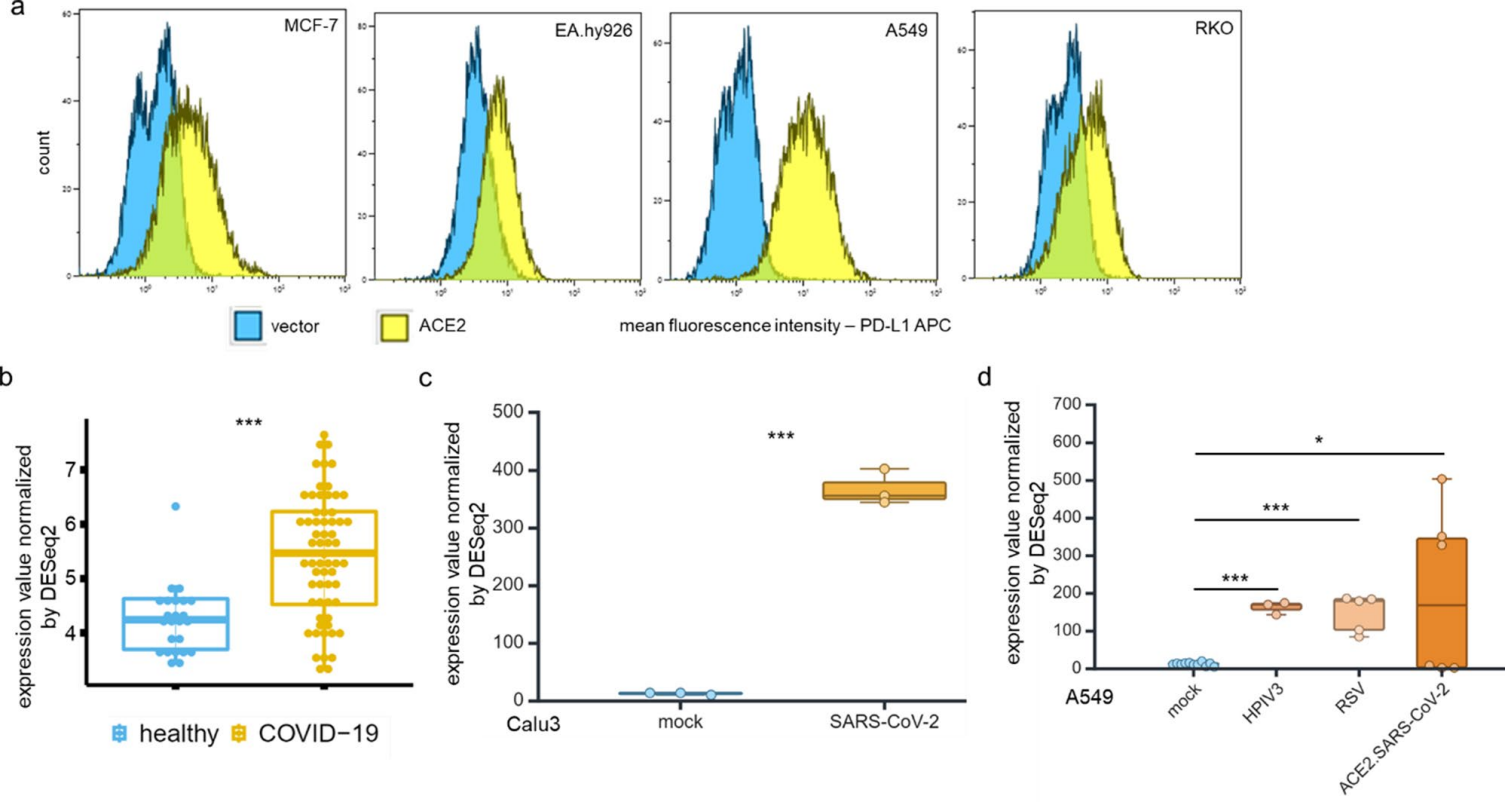
Increased PD-L1 expression of ACE2^high^ cells and respiratory viral infections. **a** The ACE2 transfectants of EA.Hy926, A549, RKO and MCF-7 cells were analyzed for PD-L1 surface expression using flow cytometry (*n* = 3). The results are shown as MFI of x-fold regulation to mock-transfected cells. **b** PD-L1 mRNA levels in blood samples of COVID-19 patients (62 COVID-19 patients vs. 24 healthy controls) **c**, **d** The levels of PD-L1 mRNA were analyzed from RNA-seq data of SARS-CoV-2-infected Calu3 (c) and ACE2^high^ A549 cells (d) and other respiratory viral infections on A549 (d) (GEO: GSE147507)

It was postulated that the ACE2-mediated upregulation of immune modulatory molecules might be associated with an increased response to immunotherapy [50], since the treatment of SARS-CoV-2-infected patients with ICPi might enhance anti-viral T cell responses by affecting PD-L1 expression [51]. Indeed, ACE2^high^ MCF-7 cells expressed higher PD-L1 levels than the ACE2^low^ control cells (Fig. 5a). This was accompanied by an increased expression of different IFN-γ signaling pathway components in ACE2^high^ EA.Hy926, A549, RKO and MCF-7 cells and is in line with the IFN-γ-mediated upregulation of PD-L1. Comparable results were retrieved from in silico data of blood samples (Log2FC 1.19; p value 4.43E-06) (Fig. 5b) and lung biopsies from COVID-19 patients compared to their healthy counterparts (Supplementary Fig. 5) as well as from cells infected by SARS-CoV-2 (Fig. 5c) or other respiratory viruses (Fig. 5d).

### ACE2high, not ACE2low MCF-7 cells, increased immune cell migration and apoptosis upon nivolumab treatment

**Fig. 6:**
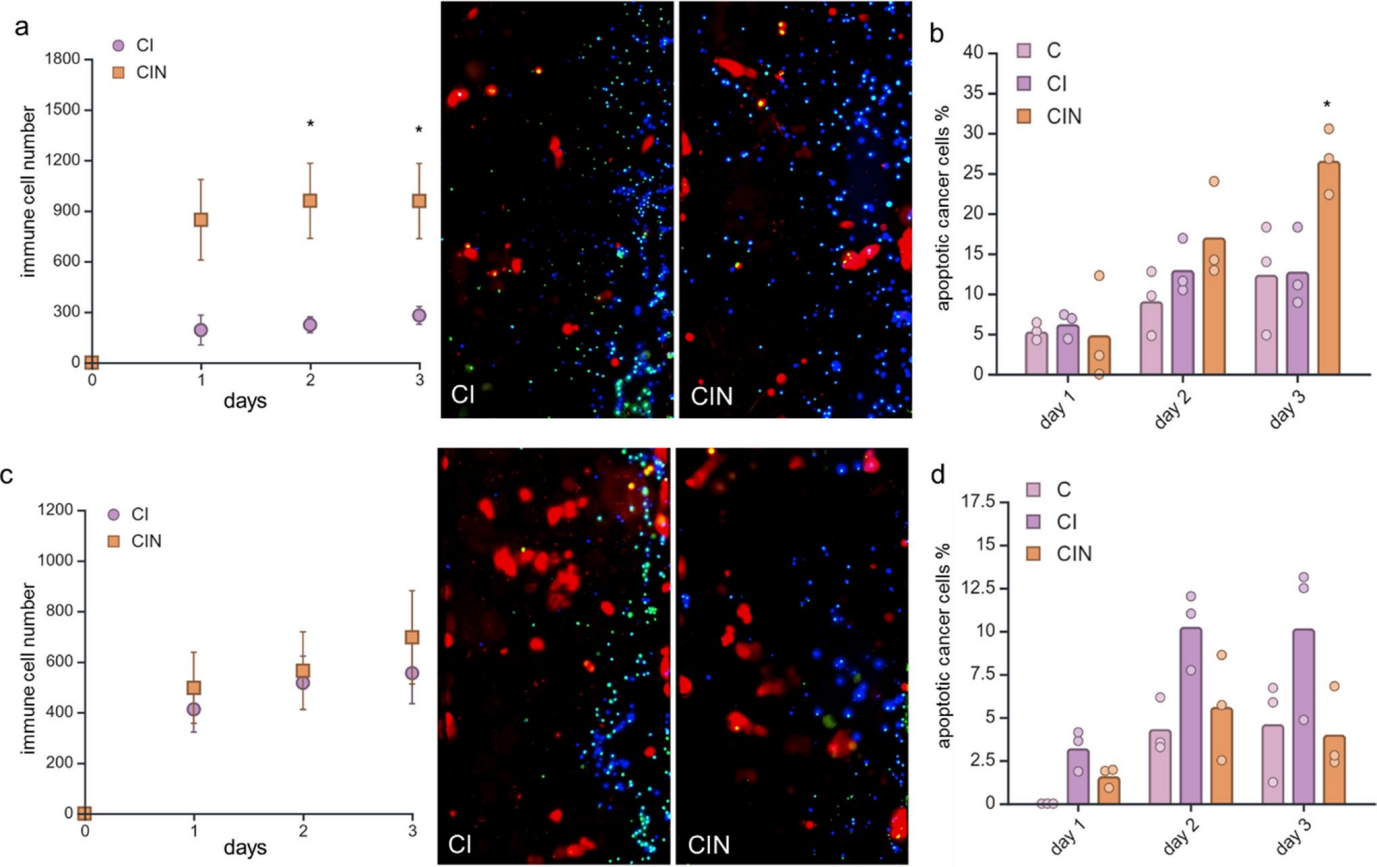
Nivolumab treatment induces migration of immune cells towards ACE2^high^ cells and apoptosis of ACE2 transfected cancer cells. **a**, **c** Over a period of 3 days, immune cells migrated notably towards ACE2^high^ MCF-7 cells treated with nivolumab (a), whereas the nivolumab treatment had no significant migration of immune cells towards ACE2^low^ cells (c). The immunofluorescent images were taken on day 3, displaying a representative image. Cancer cells are depicted in red, lymphocytes in blue, and apoptotic cells in green in the fluorescence images. **b**, **d** Percentage of apoptotic cells of ACE2^high/low^ MCF-7 cells. Nivolumab treatment of immune cells exhibited an increased apoptotic rate in ACE2^high^ cells (b) than in the untreated control but not in the ACE2^low^ cells (d). C - Cancer cells, CI - Cancer cells co-cultured with Immune cells, CIN – CI treated with nivolumab

**Fig. 7:**
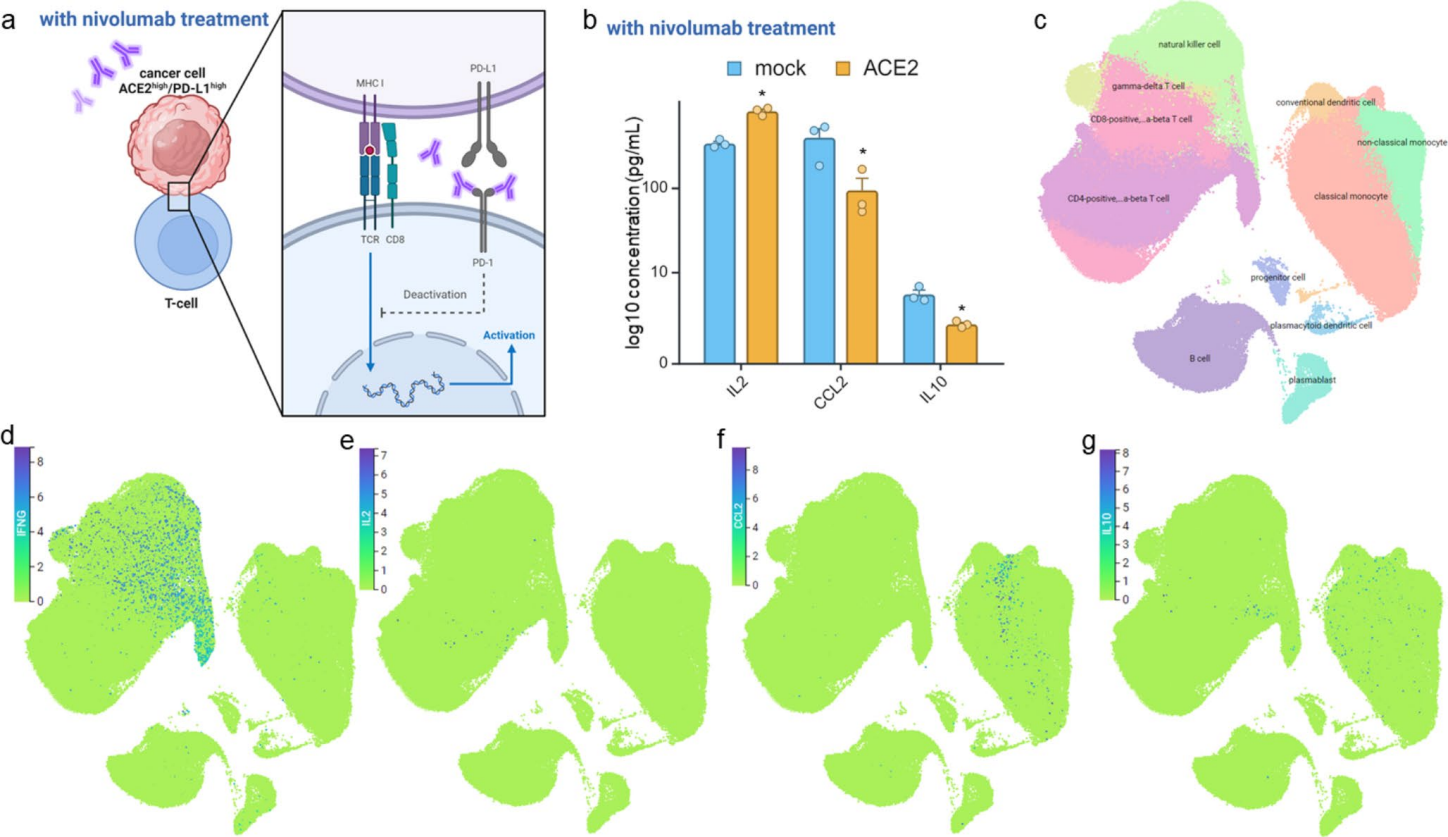
Analysis of cytokine expression in PBMNCs of COVID-19 patients and the impact of nivolumab treatment on cytokine release in ACE2^high/low^ cells. **a** Nivolumab as programmed death-1 (PD-1) inhibitor for targeted immunotherapy of tumor cells to activate T cells. **b** Nivolumab treatment significantly increased IL2 and decreased CCL2 and IL-10 release on ACE2^high^ MCF-7 cells compared to mock cells. **c** Data from scRNA-seq of PBMNCs from a cohort of 425,398 single cells (CZ CELLxGENE Discover). UMAP of various cell types, including plasmablasts, B cells, CD4^+^, CD8^+^, and γδ T cells, NK cells, conventional and plasmacytoid dendritic cells, classical and non-classical monocytes, and hematopoietic progenitor cells. **d**, **e**, **f**, **g** Higher mRNA expression levels of IFNG (d), IL2 (e), CCL2 (f) and IL10 (g) in different cell types of PBMNCs

In the next step, immune cell migration, cancer cell proliferation and apoptosis was investigated over a period of three days upon co-culturing ACE2^low/high^ MCF-7 cells with immune cells in the presence and absence of the anti-PD1 monoclonal antibody nivolumab. The PD-1 inhibitor induced significant immune cell infiltration towards ACE2^high^ MCF-7 cells (Fig. 6a), which significantly increased over time. In contrast, the number of immune cells migrating towards cancer cells was minimal in ACE2^mock/low^ MCF-7 and was influenced by nivolumab treatment (Fig. 6c). In addition, nivolumab treatment showed a tendency of increased apoptosis in ACE2^high^ (Fig. 6b), but not of ACE2^low^ MCF-7 cells (Fig. 6d). At the same time, nivolumab had neither an effect on the proliferation of ACE2^high^ nor of ACE2^low^ MCF7 cells (data not shown). The higher expression of HLA class I on ACE2^high^ MCF-7 cells might be responsible for the increased migration and apoptosis, potentially leading to the activation of T cells, while the inhibitory effect of the increased PD-L1 expression in ACE2^high^ MCF7 cells might be blocked by nivolumab (Fig. 7a).

### Association of altered cytokine release with ACE2 and PD-L1 expression upon nivolumab treatment

Despite a cytokine storm has been widely reported to be caused by viral respiratory infections of influenza viruses and SARS-CoV-2 [52, 53], it has not been directly linked to immune cells. To investigate the impact of nivolumab on cytokine release in cell supernatants of ACE2^high^ and ACE2^low^ MCF-7 cells during co-culture with PBMNCs were analyzed with a human FirePlex^®^-96 key cytokine immunoassay panel, which consists of the 17 cytokines CSF2, IL1B, IL2, IL4, IL5, IL6, CXCL8, IL9, IL10, IL12A, IL13, IL17A, IFNG, CCL2, CCL3, CCL4 and TNF. As shown in Fig. 7b, treatment of ACE2^high^ and ACE2^low^ MCF-7 cells with nivolumab decreased the release of the innate immunity-related cytokine CCL2 and increased the secretion of the adaptive immunity-related cytokine IL-2. Furthermore, the secretion of the anti-inflammatory cytokine IL-10 known to inhibit MHC class I expression was significantly reduced following nivolumab treatment (Fig. 7b), while the other cytokines analyzed did not show statistically significant changes (Supplementary Table 4A).

### Altered cytokine expression profile in peripheral blood cells and lung epithelium in COVID-19 patients

To assess the expression status of the differentially secreted cytokines upon ACE2 overexpression and treatment with nivolumab, single cell (sc) RNA-seq data from PBMNCs obtained from a cohort of 425,398 single cells from COVID-19 patients [54] were examined using CZ CELLxGENE Discover. Using the uniform manifold approximation and projection (UMAP), the expression of IFNG, IL6, IL2, IL10, and CCL2 was determined across various immune cell subtypes, including plasma blasts, B cells, CD4^+^, CD8^+^, γδ T cells, NK cells, conventional and plasmacytoid dendritic cells, classical and non-classical monocytes and hematopoietic progenitor cells (Fig. 7c). The UMAP revealed high IFNG mRNA levels in CD4^+^, CD8^+^ and γδ T cells as well as NK cells (Fig. 7d), while IL2 expression was found to be high in CD4^+^ T cells (Fig. 7e). In addition, CCL2 expression exhibited higher levels in classical monocytes and dendritic cells (Fig. 7f), while an upregulation of IL10 mRNA was detected in monocytes and CD4^+^ T cells of COVID-19 patients (Fig. 7g). Since the lung epithelium is a major target of the cytokine storm [44], a separate in silico analysis was performed on lung biopsies from severe COVID-19 patients (GEO: GSE147507) to assess the expression of these cytokines. The analysis revealed low IL2, but high CCL2 and IL10 mRNA expression (Supplementary Table 4B).

## Discussion

High ACE2 expression levels were associated with SARS-CoV-2 infection [26], but were also frequently found in various cancers [55, 56]. ACE2 exerts different activities and is involved in the modulation of oncogenic pathways and epithelial-to-mesenchymal transition (EMT), thereby affecting anti-tumor immunity and the outcome of tumor patients [50]. Our study demonstrated an impact of ACE2 on IFN signaling components in tumor cell lines and patients’ samples, which is linked to an upregulation of HLA class I, but also of PD-L1, suggesting that cancer patients with COVID-19 might have an increased response to checkpoint inhibitor treatment, such as nivolumab or durvalumab. To get further insights into the role of the ACE2-mediated increased HLA class I and PD-L1 surface expression, the next step should be to conduct in vivo studies in mice to validate the findings from our in vitro and in silico experiments in the presence and absence of ICPi and unravel its effect on anti-tumoral immune responses. In addition, CRISPR/Cas9-mediated knock down of ACE2 expression will be crucial to further support our conclusions. A decreased expression of ACE2 as well as PD-L1 (GEO: GSE165025) was found after CRISPRi knockdown of BRD2, another key molecule in host–SARS–CoV–2 interaction in Calu-3 cells [57]. This finding further supports the link between ACE2 and PD-L1 in the SARS-CoV-2 context.

Indeed, in silico analyses of TCGA data revealed a significantly increased expression of ACE2 and of IFN-regulated molecules, such as MHC class I APM components and PD-L1, in COVID-19 patients when compared to their controls, which is in line with the COVID-19 genome databases. Moreover, GO and KEGG enrichment analyses indicated an enrichment of several signaling pathways associated with anti-viral immunity, which were described in COVID-19 disease [58–60]. SARS-CoV-2 induces a more robust IFN-I response than SARS-CoV-1 in human cells [61, 62]. The IFN-α-inducible protein 27 (IFI27) was the top upregulated gene in the COVID-19 cohort [43], which is an early predictor for the outcome of COVID-19 patients [63, 64]. In line with these data, our ACE-2^high^ MCF-7 transfectants expressed increased IFI27mRNA levels (Log2FC 6.47; p 2.38E-148).

It is well known that IFN could induce the major HLA class I APM component expression in tumor cells [65]. Although many reports discussed the ACE2-mediated induction of IFN pathway, APM components were less studied in the context of ACE2 overexpression or coronavirus infection. In this study, higher APM component expression including TAP1 was found in in vitro models of ACE2^high^ cells and in in silico COVID-19 datasets. These findings are in line with higher TAP1 levels described in the lung epithelia upon SARS-CoV-2 infection of ACE2 mice [66].

Growth factors and inflammatory cytokines, such as EGF, IL-6, IFN-γ, TNF-α and TGF-β, can induce PD-L1 expression [67, 68]. It remains unclear whether anti-PD-1/anti-PD-L1 treatment would benefit COVID-19 patients, since ICPi treatment might enhance the cytokine storm associated with higher COVID-19 morbidity and mortality [69, 70]. However, melanoma patients suffering from COVID-19 displayed better outcomes upon ICPi co-treatment [71, 72]. Patients with metastatic squamous head and neck cancer treated with ICPi had an increased risk of recall immune-mediated pneumonitis upon SARS-CoV-2 infection [73], while the incidence of serious adverse events upon ICPi and chemotherapy treatment was higher for SARS-CoV-2-positive patients [74]. Blocking PD-L1 in mice infected with AAV-hACE2 significantly recovered lymphocyte counts and lowered inflammatory cytokine levels [68]. Furthermore, the anti-PD-L1 therapy was associated with a reduced neutrophil-to-lymphocyte ratio, which is beneficial for the overall survival of renal cell cancer and non-small-cell lung carcinoma patients [68, 75]. Therefore, the anti-PD-L1 therapy might also benefit SARS-CoV-2-infected patients, despite studies are needed to dissect how anti-PD-L1 therapy affects ACE2, cytokine storm, IFN signaling and lymphocyte composition and function following SARS-CoV-2 infection. ACE2^high^ cells responded to nivolumab by increasing the immune cell infiltration and inducing apoptosis of cancer cells in an ACE2-dependent manner (Fig. 6).

Furthermore, decreased innate immunity and the release of anti-inflammatory cytokines CCL2 and IL10 were detected in ACE2 transfectants upon nivolumab treatment in vitro. This CCL2 and IL10 decline might revert the T cell exclusion and cytokine storm in ICPi-resistant “cold tumors with COVID-19 infection” in vivo. Cytokines can mediate the expansion, activation and trafficking of effector lymphocytes, but can also recruit regulatory T cells (Treg) [76]. IL-10 can block NF-κB activity, while interaction between IL-10 and its receptor activates the JAK-STAT signaling pathway [77, 78]. IL10 and CCL2 are the most prominent cytokines predicting COVID-19 severity [75], but also higher levels of IL2, IL6, IL7, IL10, CXCL10, CCL2, TNF, macrophage inflammatory protein 1 alpha, type-I IFN, and CSF2 were reported in the serum or plasma of patients with severe COVID-19 than in patients with mild and moderate infections [2, 34, 79, 80] supporting the evidence of a cytokine storm [81]. In a study conducted by D’Addio and co-authors [82], it was shown that the SARS-CoV-2 mRNA vaccine may lead to a reduced cellular immune response specific to SARS-CoV-2, particularly in relation to IL2, in individuals with type 1 diabetes. This highlights the need to further explore the effects of COVID-19 vaccines and ICPi treatments in future.

**Fig. 8:**
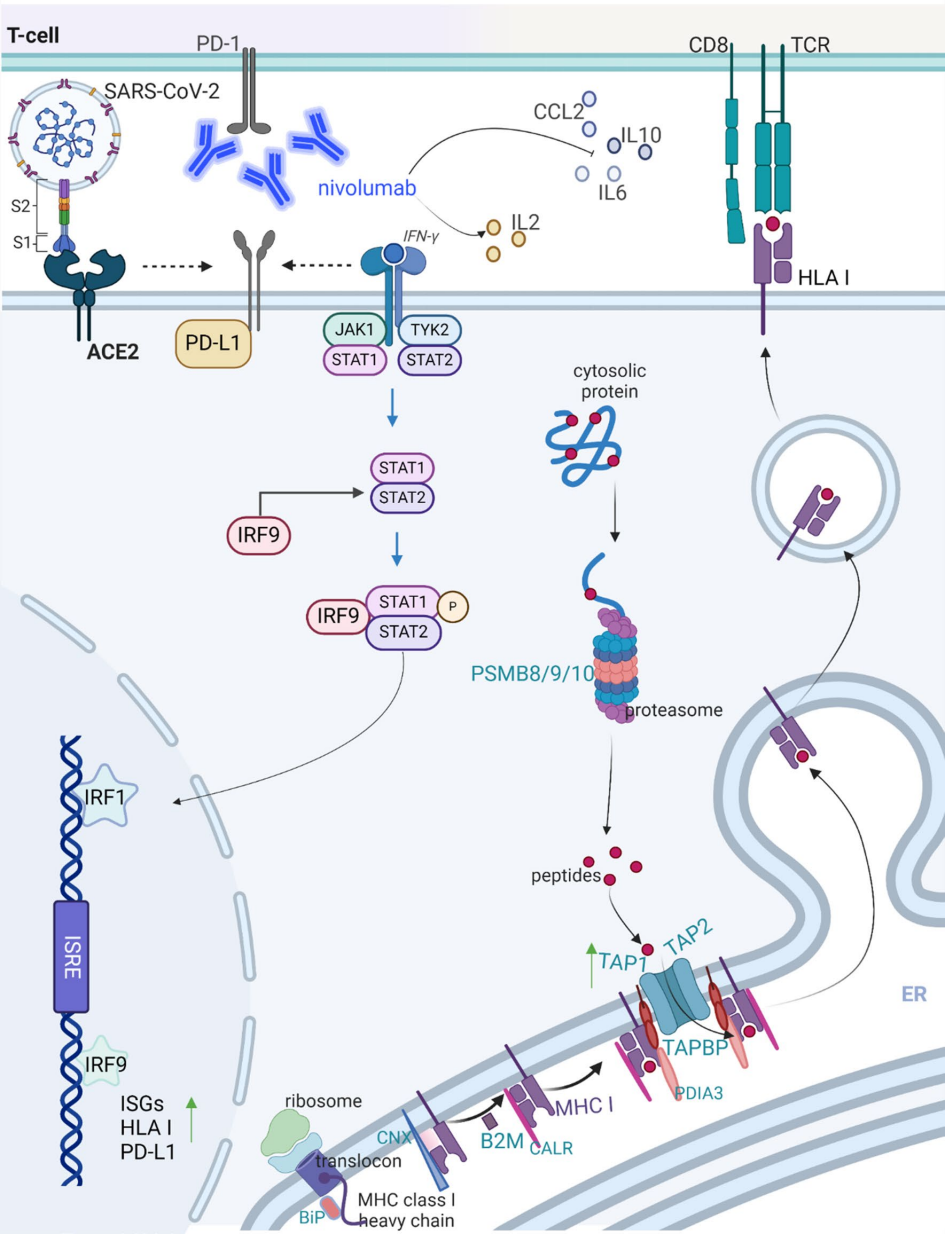
The cells with high levels of ACE2 showed higher levels of PD-L1, the IFN pathway, HLA class I APM, and immune responses compared to the cells with low levels of ACE2. This suggests that ACE2 expression, in different model systems and after SARS-CoV-2 infection, is associated with changes in host immunogenicity. These changes could potentially impact the effectiveness of the PD-1 inhibitor nivolumab, which increased immune cell migration and reduced cytokines related to innate immunity (created with BioRender.com)

Cytokine release via activation of the JAK/STAT signaling pathway following SARS-Cov-2 infection resulting in acute respiratory distress syndrome related to COVID-19 [83] and IFN-JAK-STAT pathway components were higher in ACE2^high^ cells (Fig. 8). Mice producing an early strong IFN response to SARS-CoV-2 were likely to live, but in other cases, the disease progressed to a highly morbid, overactive immune system [84]. In addition to their potential therapeutic value, cytokines could activate the effector lymphocytes after the initiation of ICPi treatment [85]. The secretion of CCL2 and IL10 was significantly downregulated after nivolumab treatment of ACE2^high^ MCF-7 cells compared to controls (Fig. 7b). Clinical trials employed combination therapy of cytokine blockade and ICIs for different cancers [76]. Due to the increased levels of cytokines, including IFNs in patients with severe COVID-19, both have been investigated as potential targets for SARS-CoV-2 therapy.

## Conclusions

Our research emphasizes the importance of using in vitro ACE2-based disease models and RNA-seq datasets to form hypotheses relevant to human disease. With the emergence of new SARS-CoV-2 variants, research should prioritize efficient targeting and therapeutic strategies aimed at ACE2, the common entry point for a wide range of viruses, including different coronavirus species. Further research is needed to understand the impact of SARS-CoV-2 on ACE2 expression and the timing of host responses at the single-cell level and identify potential host factors influencing these processes. Our findings show that treatment with an anti-PD-1 antibody promotes immune cell infiltration and reduces the production of inflammatory cytokines in an in vitro ACE2^high^ model suggesting that iCPi treatment may alleviate T cell exhaustion and hinder virus infectivity at the early stage of virus entry [68, 86]. Surveillance and prevention remain the most effective measures to counter potential new outbreaks due to the complex interaction between humans and coronaviruses.

## Electronic supplementary material

Below is the link to the electronic supplementary material.

Supplementary Material 1

## Data Availability

All data generated or analyzed during this study are included either in this article or in the supplementary information files. The datasets generated during and/or analyzed during the current study are available from the corresponding author on reasonable request.

## Abbreviations

Ab: Antibody
ACE2: Angiotensin-converting enzyme-2
APM: Antigen processing machinery
B2M: β_2_-microglobulin
BP: Biological process
CC: Cellular component
CoV: Coronavirus
CSF: Colony stimulating factor
DDS: Disease-specific survival
DEG: Differentially expressed gene
FCS: Fetal calf serum
GADPH: Glyceride aldehyd-3-phosphate dehydrogenase
GO: Gene ontology
HLA: Human leukocyte antigen
HPIV3: Human parainfluenza virus 3
HRP: Horse reddish peroxidase
IAVdNS: Mutant influenza A virus
ICPi: Immune checkpoint inhibitor
IFN: Interferon
IL: Interleukin
mAb: Monoclonal antibody
MCP-1: Monocyte chemoattractant protein-1
MF: Molecular function
MFI: Mean fluorescence intensity
MHC: Major histocompatibility complex
MIP-1α: Macrophage inflammatory protein 1 alpha
NK: Natural killer
OS: Overall survival
PBMNC: Peripheral blood mononuclear cells
PD1: Programed death receptor 1
PD-L1: Programed death ligand 1
RNA-seq: RNA sequencing
RSV: Respiratory syncytial virus
S: Spike
SARS: Severe acute respiratory syndrome
TAP: Transporter associated with antigen processing
TCGA: The Cancer Genome Atlas
TGF-β: Transforming growth factorβ
TME: Tumor microenvironment
TNF: Tumor necrosis factor
