# Supplementary material for: Altered ACE2 and interferon landscape in the COVID-19 microenvironment correlate with the anti-PD-1 response in solid tumors
Source: Cell Mol Life Sci. 2024 Dec 3;81(1):473. doi: 10.1007/s00018-024-05520-9 (PMC11615173; doi:10.1007/s00018-024-05520-9)

Supplementary Table 1: Primers used for qPCR analyses.

| gene | forward | reverse |
|---|---|---|
| ACE2 | GGGATCAGAGATCGGAAGAAGAAA | AGGAGGTCTGAACATCATCAGTG |
| HLA-ABC | GCCTACCACGGCAAGGATTAC | GGTGGCCTCATGGTCAGAGA |
| CD274 | AAGGGAGAATGATGGATG | ATTCTCCTCCTCTGCTTT |
| TAP1 | GGAATCTCTGGCAAAGTCCA | TGGGTGAACTGCATCTGGTA |
| TAP2 | CCAAGACGTCTCCTTTGCAT | TTCATCCAGCAGCACCTGTC |
| TAPBP | TGGGTAAGGGACATCTGCTC | ACCTGTCCTTGCAGGTATGG |
| PSMB9 | TGCTGCATCCACATAACCAT | TGTGCACTCTCTGGTTCAGC |
| PSMB8 | TCTGCGTCATCAGCAAGAAC | GCCATTCAGGAAGTGTCCAT |
| PSMB10 | GGGCTTCTCCTTCGAGAACT | CAGCCCCACAGCAGTAGATT |
| B2M | CTCGCGCTACTCTCTCTT | AAGACCAGTCCTTGCTGA |
| ERAP1 | TCATCCTCCCAGAAGAGGTG | AACCTTGAAACACGGGCATA |
| CANX | TGTGAGTCAGCTCCTGGATG | GACCACAGCTCCAAACCAAT |
| CALR | TGGACCCGATATTTGTGGA | TCCCCAATCCTTCGATTCTG |
| IRF1 | GCAGCTACACAGTTCCAGG | GTCCTCAGGTAATTTCCCTTCCT |
| IRF2 | TGCGGTCCTTGACTTCAACTA | ATATTCCTCTTCCGCCAGTG |
| IRF9 | GTCCAGCTGTCTGGAAGACT | TCCTCTTCCTCCTTCCTCTC |
| JAK1 | CCACTACCGGATGAGGTTCTA | GGGTCTCGAATAGGAGCCAG |
| JAK2 | TCTGGGGAGTATGTTGCAGAA | AGACATGGTTGGGTGGATACC |
| TYK2 | GAGATGCAAGCCTGATGCTAT | GGTTCCCGAGGATTCATGCC |
| STAT1 | CAGCTTGACTCAAAATTCCTGGA | TGAAGATTACGCTTGCTTTTCCT |
| STAT2 | GAGCCAGCAACATGAGATTGA | GCCTGGATCTTATATCGGAAGCA |
| STAT3 | ATCACGCCTTCTACAGACTGC | CATCCTGGAGATTCTCTACCACT |
| STAT6 | CGAGTAGGGGAGATCCACCTT | GCAGGAGTTTCTATCAAGCTGTG |
| STING | GTCTCCAAGCCTCTGGACTG | TGTCAAGTCCTGGACCCTTC |
| IFI6 | GGTCTGCGATCCTGAATGGG | TCACTATCGAGATACTTGTGGGT |
| IFIT1 | TTGATGACGATGAAATGCCTGA | CAGGTCACCAGACTCCTCAC |
| IFIT2 | AAGCACCTCAAAGGGCAAAAC | TCGGCCCATGTGATAGTAGAC |
| IFIT3 | AACAGATGTCCTCCGCAGTG | TTCCGTCTCCAGGAACTCAG |
| OAS2 | GACACTGCAGAAGGTTTCCG | CTGCATTGTCGGCACTTTCC |
| OASL | CCATTGTGCCTGCCTACAGAG | CTTCAGCTTAGTTGGCCGATG |
| HLA-B | CAGTTCGTGAGGTTCGACAG | CAGCCGTACATGCTCTGGA |
| OAS1 | GGGATTTCGGACGGTCTTGG | TCGTCGGTCTCATCGTCTGC |
| DDX60 | GACAGGTCCAGGTGTCAGTTT | TTGGTCTCGTGTGCCTTTTTG |
| CMPK2 | CCAGGTTGTTGCCATCGAAG | CAAGAGGGTGGTGACTTTAAGAG |
| SCD | TCTAGCTCCTATACCACCACCA | TCGTCTCCAACTTATCTCCTCC |
| ABCG1 | ATTCAGGGACCTTTCCTATTCGG | CTCACCACTATTGAACTTCCCG |
| SREBF1 | GCCCCTGTAACGACCACTG | CAGCGAGTCTGCCTTGATG |
| FGFR4 | CGACACAAGAACATCATCAACC | CATCACATTGTCCTCAGTCACC |
| PHGDH | CTGCGGAAAGTGCTCATCAGT | TGGCAGAGCGAACAATAAGGC |
| FBXO27 | ACAGACTCCTCGTCCAACTTC | GGCAGGCATTGTTGTTCCAC |
| PREX1 | GGCATTCCTGCATCGCATC | CGGGTGTAAACAATACTCCAAGG |
| CRAT | GTGGCTCAAGACCGCCTAC | GCAGCAAATCGGAGCTGAC |
| AIF1L | GGTGCTTCCTGCACAGAGAT | GACCGTTTCCCCAGCATCAT |
| PXMP4 | CCCCCATCACGCCTTCTAAA | GGAGGGCACAGGGAGTTTTC |
| ACTB | ACTCTTCCAGCCTTCCTTCC | AGCACTGTGTTGGCGTACAG |
| GAPDH | CTGGTAAAGTGGATATTGTTGCCAT | TGGAATCATATTGGAACATGTAAACC |

Supplementary Table 2: DEGs linked to different types of diseases. Top DEGs annotated to each of these diseases are presented.

| gene_name | disease_id |
|---|---|
| IFI6 | C0000786,C0011311,C0019196,C0021400,C0023903,C0024623,C0026764,C0033860,C0042769,C0596263,C0699791,C1140680,C2239176 |
| IFIT1 | C0004114,C0014175,C0019196,C0021400,C0024141,C0024623,C0027092,C0030389,C0036421,C0042769,C0206633,C0282687,C0699791,C1140680,C3463824,C3714514 |
| IFIT2 | C0007137,C0021400,C0025202,C0035235,C0043124,C1096184,C1319315,C1621958,C3714514 |
| IFIT3 | C0002395,C0004364,C0008497,C0013080,C0021400,C0022104,C0023418,C0023530,C0024141,C0027627,C0235974,C0346647,C0524851,C1458155 |
| OAS2 | C0007682,C0008055,C0011311,C0011570,C0011581,C0011615,C0011854,C0013595,C0014061,C0019163,C0019196,C0021400,C0025289,C0033860,C0036421,C0041696,C0042769,C0153381,C0220641,C0282687,C0338715,C0376358,C0524909,C0600139,C0936250,C1269683 |
| OASL | C0014061,C0019196,C0021400,C0024141,C0036421,C0040336,C0375023,C0524909,C0524910,C1096184 |
| HLA-B | C0000737,C0000809,C0000832,C0000887,C0001175,C0001403,C0001418,C0001627,C0001824,C0001973,C0002171,C0002390,C0002395,C0002736,C0002871,C0002874,C0002902,C0002940,C0003123,C0003493,C0003838,C0003862,C0003864,C0003872,C0003873,C0004096,C0004352,C0004364,C0004565,C0004623,C0004763,C0004943,C0005411,C0005586,C0006118,C0006142,C0006267,C0006309,C0006413,C0007102,C0007130,C0007131,C0007134,C0007137,C0007194,C0007222,C0007570,C0007847,C0007873,C0007930,C0008031,C0008313,C0008533,C0008728,C0009324,C0009402,C0009447,C0009676,C0009763,C0010051,C0010346,C0010418,C0010823,C0011168,C0011311,C0011334,C0011603,C0011609,C0011615,C0011633,C0011644,C0011847,C0011849,C0011854,C0011860,C0011991,C0013080,C0013132,C0013182,C0013221,C0013264,C0013295,C0013421,C0014070,C0014175,C0014457,C0014518,C0014544,C0014661,C0014743,C0014859,C0015230,C0015397,C0015405,C0015672,C0015696,C0015967,C0016059,C0016382,C0017086,C0017152,C0017160,C0017168,C0017178,C0017181,C0017636,C0017638,C0017661,C0017677,C0017980,C0018099,C0018133,C0018213,C0018522,C0018621,C0018790,C0018801,C0018939,C0018989,C0018995,C0019050,C0019069,C0019100,C0019101,C0019114,C0019163,C0019187,C0019196,C0019360,C0019655,C0019693,C0020097,C0020456,C0020458,C0020517,C0020538,C0020 |

| | |
|---|---|
| | 542,C0020546,C0020676,C0021345,C0021368,C0021390,C0021400,C0022104,C0022408,C0022548,C0022658,C0022661,C0022972,C0023343,C0023364,C0023418,C0023434,C0023467,C0023470,C0023473,C0023493,C0023787,C0023890,C0023891,C0023892,C0023903,C0024115,C0024121,C0024137,C0024141,C0024299,C0024305,C0024314,C0024530,C0024534,C0024535,C0025007,C0025202,C0025289,C0026654,C0026691,C0026769,C0026780,C0026896,C0026916,C0026948,C0027051,C0027121,C0027339,C0027430,C0027439,C0027498,C0027627,C0027643,C0027686,C0027697,C0027721,C0027794,C0027796,C0027813,C0027819,C0027873,C0028797,C0029134,C0029172,C0029191,C0029408,C0029458,C0029899,C0030297,C0030319,C0030481,C0030567,C0030807,C0030809,C0030848,C0031022,C0031099,C0031106,C0031762,C0032026,C0032227,C0032768,C0032787,C0032962,C0033687,C0033770,C0033860,C0033975,C0034069,C0034150,C0034152,C0034362,C0034902,C0035012,C0035078,C0035243,C0035335,C0035357,C0035435,C0035439,C0035455,C0036202,C0036220,C0036341,C0036391,C0036421,C0036572,C0036690,C0037036,C0037116,C0037274,C0037286,C0037299,C0037928,C0038012,C0038013,C0038325,C0038356,C0038363,C0038579,C0038990,C0039103,C0039263,C0039483,C0039520,C0040038,C0040147,C0040561,C0040592,C0041234,C0041296,C0041324,C0041326,C0041327,C0041374,C0041466,C0041755,C0041834,C0042109,C0042111,C0042164,C0042165,C0042170,C0042344,C0042384,C0042487,C0042749,C0042769,C0042900,C0079487,C0079731,C0085106,C0085183,C0085207,C0085409,C0085413,C0085435,C0085580,C0085636,C0085669,C0085786,C0085932,C0086543,C0087031,C0149521,C0149678,C0149745,C0149778,C0149871,C0149925,C0149931,C0151281,C0151786,C0151811,C0152018,C0155118,C0162119,C0162526,C0162538,C0162539,C0162566,C0162871,C0175708,C0206062,C0206114,C0206138,C0206178,C0206708,C0221259,C0231528,C0235974,C0237653,C0238301,C0238463,C0241054,C0241128,C0241910,C0242379,C0242383,C0242422,C0242510,C0242994,C0243026,C0263361,C0264939,C0266929,C0268074,C0268318,C0271737,C0275524,C0276501,C0278883,C0278996,C0282193,C0302592,C0332563,C0332573,C0332996,C0342342,C0343752,C0345049,C0345905,C0346647,C0348801,C0376358,C0376545,C0392514,C0393593,C0398650,C0400936,C0409667,C0409974,C0423463,C0427515,C0458219,C0497169,C0520463,C0521707,C0524702,C0524909,C0524910,C0543698,C0546837,C0553580,C0553730,C0566602,C0575081,C0595921,C0600139,C0677607,C0677932,C0678222,C0684249,C0687675,C0699790,C0700590,C0701807,C0702166,C0741395,C0744641,C0746926,C0751356,C0751713,C0751911,C0751967,C0752160,C0752262,C0850666,C0851140,C0852654,C0856825,C0858318,C0860207,C0867389,C0878555,C0887976,C0919267,C0920028,C0920350,C0948008,C0948192,C0948954,C0949690,C0949691,C0950124,C1096184,C1140680,C1142253,C1168198,C1175175,C1262477,C1264606,C1274216,C1274933,C1290884,C1302773,C1304140,C1306460,C1306759,C1328252,C1334177,C1367654,C1402315,C1456792,C1458155,C1510420,C1510586,C1519176,C1527249,C1527336,C1565489,C1621958,C1719495,C1720830,C1800706,C1832588,C1837388,C1847835,C1849193,C1868649,C1956346,C1956391,C1960272,C1961102,C1962972,C2103602,C2132198,C2220104,C2239176,C2316810,C2363973,C2919828,C2931822,C2936858,C2937365,C2939465,C2973725,C3164445,C3203102,C3241919,C3276706,C3 |

| | |
|---|---|
| | 469186,C3495436,C3495801,C3539781,C3658302,C3662483,C3697982,C3714514,C3854222,C3887505,C4020848,C4020887,C4020969,C4048328,C4277682 |
| OAS1 | C0002871,C0004096,C0007102,C0008055,C0010674,C0011311,C0011854,C0014061,C0018572,C0019163,C0019196,C0021400,C0023890,C0025202,C0026769,C0042769,C0043124,C0239946,C0376358,C0524909,C0524910,C0600139,C0699790,C0751583,C1096184,C1140680,C1175175,C1527390,C3714514,C4277682 |
| DDX60 | C0021400,C0596263 |
| CMPK2 | C0007847,C0017638,C0019693,C0021364,C0022521,C0023418,C0023448,C0023449,C0023473,C0023895,C0025202,C0027627,C0027819,C0035243,C0040336,C0041296,C0206708,C0302592,C0549473,C0600139,C0678222,C0686619,C0700095,C1527249,C1961102,C3854222,C4048328 |
| SCD | C0001418,C0002170,C0002395,C0002726,C0002736,C0002895,C0003130,C0003850,C0004096,C0004135,C0004153,C0005283,C0005684,C0005695,C0005859,C0006142,C0007102,C0007112,C0007222,C0007621,C0008626,C0009375,C0009402,C0010054,C0010068,C0011847,C0011849,C0011854,C0011860,C0011881,C0015397,C0015695,C0018213,C0018790,C0018801,C0018802,C0019045,C0020443,C0020459,C0020474,C0020557,C0021294,C0021655,C0023223,C0023449,C0023794,C0023903,C0023976,C0024623,C0024667,C0024668,C0025517,C0026946,C0027627,C0028754,C0028756,C0029422,C0029925,C0030193,C0032460,C0033578,C0035828,C0036341,C0037054,C0037274,C0039070,C0040136,C0043094,C0178874,C0220656,C0234233,C0235950,C0238461,C0242339,C0242379,C0271650,C0275524,C0278488,C0278996,C0279702,C0280100,C0333516,C0339143,C0376358,C0410528,C0410787,C0521158,C0524620,C0549473,C0596263,C0600139,C0678222,C0684249,C0699790,C0699791,C0699885,C0741923,C0878544,C1140680,C1141890,C1168401,C1263846,C1269683,C1306460,C1458155,C1527249,C1851585,C1859486,C1956346,C2239176,C2711227,C3150943,C3841475,C3887505 |
| ABCG1 | C0002395,C0002726,C0003850,C0004153,C0005586,C0006142,C0007131,C0007222,C0009402,C0009404,C0010054,C0010068,C0011847,C0011849,C0011860,C0013080,C0013537,C0020443,C0020473,C0022661,C0023794,C0023893,C0024115,C0025500,C0027022,C0027051,C0027404,C0028754,C0031117,C0034050,C0034069,C0041107,C0042373,C0043094,C0151744,C0242383,C0376358,C0524620,C0525045,C0600139,C0678222,C0745103,C0856169,C1292778,C1335302,C1527249,C1709246,C1839839,C1852197,C1863340,C1956346,C1970943,C1970945,C2613439,C2700438,C2700439,C2700440,C3658248 |
| SREBF1 | C0002395,C0003130,C0003504,C0003507,C0003850,C0004153,C0006142,C0007103,C0007124,C0009402,C0010054,C0010068,C0010823,C0011265,C0011847,C0011849,C0011853,C0011854,C0011860,C0013274,C0015695,C0015814,C0017636,C0019163,C0019196,C0020443,C0020459,C0020473,C0020476,C0020538,C0020557,C0020615,C0021655,C0022638,C0022661,C0023787,C0023827,C0023893,C0023903,C0025517,C0026837 |

| | |
|---|---|
| | ,C0027627,C0028754,C0029445,C0030567,C0033300,C0033578,C0036341,C0040822,C0043094,C0079731,C0178874,C0206064,C0206669,C0220989,C0235974,C0239946,C0242339,C0263420,C0271694,C0342907,C0346647,C0376358,C0400966,C0410189,C0476089,C0497327,C0524620,C0596263,C0600139,C0678222,C0740447,C0795864,C1257763,C1280433,C1335302,C1458155,C1527249,C1883486,C1955934,C1956346,C2239176,C2711227,C2911647,C2936179,C3887505 |
| FGFR4 | C0001418,C0001430,C0001973,C0004114,C0004763,C0005684,C0005695,C0006142,C0007102,C0007114,C0007115,C0007117,C0007131,C0007134,C0007137,C0009375,C0009402,C0010278,C0010606,C0011847,C0011849,C0011860,C0011991,C0014173,C0017185,C0017636,C0018671,C0019163,C0019693,C0020437,C0020523,C0020538,C0021400,C0022104,C0023418,C0023434,C0023440,C0023449,C0023467,C0023470,C0023473,C0023474,C0023890,C0023903,C0024121,C0024299,C0024301,C0024305,C0024314,C0024623,C0025202,C0025286,C0026640,C0026764,C0027022,C0027627,C0027819,C0028754,C0029408,C0029454,C0029463,C0029925,C0030567,C0032000,C0032002,C0032019,C0032460,C0032584,C0032927,C0033578,C0035222,C0035412,C0037286,C0040136,C0079731,C0079774,C0085129,C0149721,C0149782,C0151779,C0152013,C0153676,C0175695,C0175702,C0178874,C0206180,C0206650,C0206655,C0206656,C0206659,C0206663,C0221026,C0221406,C0235974,C0238198,C0242379,C0265783,C0278493,C0278883,C0278996,C0279530,C0279628,C0280100,C0281361,C0332996,C0334576,C0334579,C0341439,C0346302,C0346647,C0376358,C0392514,C0398623,C0409952,C0476089,C0494165,C0524851,C0549473,C0549523,C0553694,C0555198,C0585362,C0596263,C0600139,C0677886,C0678222,C0684249,C0686619,C0699790,C0699791,C0699885,C0700095,C0746883,C0851887,C0853879,C0919267,C1134719,C1140680,C1168401,C1261473,C1292769,C1301034,C1301700,C1306214,C1306460,C1321422,C1321872,C1335302,C1337011,C1378703,C1384584,C1458155,C1512409,C1527249,C1561643,C1621958,C1704272,C1762616,C1961099,C2239176,C2937421,C3811653,C3887461,C4016099 |
| PHGDH | C0002736,C0002888,C0004364,C0005699,C0006118,C0006142,C0006413,C0007131,C0007621,C0007785,C0008924,C0008925,C0008928,C0009376,C0009404,C0009691,C0010417,C0010964,C0013274,C0014544,C0015300,C0015934,C0016522,C0017638,C0018817,C0018818,C0019348,C0020179,C0020224,C0020225,C0020534,C0021296,C0021841,C0022548,C0022876,C0023418,C0023473,C0024131,C0024138,C0024141,C0025202,C0025362,C0025958,C0025990,C0025995,C0026010,C0027627,C0028738,C0029463,C0029925,C0032580,C0033578,C0033999,C0036341,C0036572,C0040034,C0042769,C0080178,C0086543,C0151686,C0152421,C0158113,C0175754,C0221352,C0235659,C0240295,C0240912,C0241355,C0265218,C0265610,C0265660,C0265783,C0266463,C0266470,C0266574,C0266786,C0278701,C0279672,C0338597,C0392386,C0398791,C0409974,C0409979,C0423903,C0424605,C0424688,C0426970,C0456070,C0521525,C0542519,C0554972,C0557874,C0566694,C0580190,C0585442,C0595939,C0596263,C0678222,C0684276,C0685409,C0796195,C0850666,C0878787,C0917798,C0917816,C1140680,C1301034,C1386048,C1458155,C1510497,C1835581,C1836189,C1837385,C1837760,C1839739,C1840264,C1843005,C1843367,C1848490,C1848570,C1850189,C1850327,C1850533,C1850534,C1854885,C1855062,C1857130,C1857679,C1860450,C1860838,C1862425,C1866174,C1879312,C2053437,C2677180,C2945759,C2981 |

| | |
|---|---|
| | 150,C3277059,C3536741,C3552463,C3554617,C3714756,C3854222,C4020749,C4020757,C4020810,C4020876,C4020899,C4280304,C4280625,C4280626,C4280627,C4280808 |
| PREX1 | C0004352,C0006142,C0011860,C0022660,C0025202,C0027627,C0040336,C0162429,C0178874,C0376358,C0596263,C0600139,C0678222,C0854917,C0936223,C1282496,C1458155,C1512981 |
| CRAT | C0006142,C0007102,C0007873,C0009402,C0017921,C0018802,C0027404,C0029925,C0041327,C0085580,C0271650,C0376358,C0524620,C0600139,C0678222,C0699790,C1458155,C1527249,C3463824 |
| PXMP4 | C0178874,C0376358,C0600139 |

Supplementary Table 3: Correlation of ACE2 expression with HLA class I and APM components and IFN pathway components were performed using R2 Genomics (http://r2.amc.nl) and two different cancer datasets, Tumor Breast Invasive Carcinoma - TCGA – 1097 and Mixed Cancer GDC - TCGA – 11003.

| | **Tumor Breast Invasive Carcinoma - TCGA - 1097** | | **Mixed Cancer GDC - TCGA - 11003** | |
|---|---|---|---|---|
| | r-value | p-value | r-value | p-value |
| HLA-A | 0.198 | 3.93e-11 | 0.278 | 1.26E-194 |
| HLA-B | 0.214 | 7.79e-13 | 0.249 | 9.35E-156 |
| HLA-C | 0.136 | 5.85e-06 | 0.227 | 6.8E-129 |
| HLA-E | 0.276 | 1.30e-20 | 0.256 | 3.95E-164 |
| HLA-G | 0.128 | 2.23e-05 | 0.296 | 4.85E-222 |
| TAP1 | 0.246 | 1.47e-16 | 0.19 | 2.51E-90 |
| TAP2 | 0.290 | 1.20e-22 | 0.092 | 4.87E-22 |
| TAPBP | 0.144 | 1.80e-06 | 0.217 | 4.79E-117 |
| CALR | 0.252 | 2.66e-17 | 0.006 | 0.546 |
| CANX | -0.063 | 0.036 | 0.107 | 2.82E-29 |
| ERAP1 | 0 | 1 | 0.244 | 1.3E-148 |
| ERAP2 | 0.045 | 0.136 | 0.111 | 1.46E-31 |
| PSMB9 | 0.207 | 4.71e-12 | 0.232 | 1.25E-134 |
| PSMB8 | 0.128 | 2.26e-05 | 0.281 | 1.71E-198 |
| PSMB10 | 0.136 | 6.06e-06 | 0.254 | 2.55E-161 |
| CD274 | 0.169 | 1.96e-08 | 0.084 | 7.97E-19 |
| B2M | 0.152 | 4.03e-07 | 0.206 | 1.56E-105 |
| IFNG | 0.185 | 7.12e-10 | 0.036 | 0.00017 |
| IFNGR1 | 0.376 | 4.36e-38 | 0.187 | 8.63E-87 |
| IFNGR2 | 0.250 | 4.96e-17 | 0.076 | 1.16E-15 |
| JAK1 | 0.114 | 1.61e-04 | 0.247 | 2.38E-152 |
| JAK2 | 0.158 | 1.45e-07 | 0.045 | 0.00000205 |
| STAT1 | 0.141 | 2.87e-06 | 0.076 | 1.17E-15 |
| IRF1 | 0.206 | 6.32e-12 | 0.186 | 1.71E-86 |

Supplementary Table 4:

A: Analysis of cytokine release with ACE2$^{high/low}$ cells upon nivolumab treatment and altered cytokine expression profile in PBMNCs upon SARS-CoV-2 infection.

| | ACE2$^{low}$ | ACE2$^{high}$ | ACE2$^{high\ vs.\ low}$ |
|---|---|---|---|
| cytokines | mean | | p value |
| CSF2 | 4.18 | 4.09 | 0.47 |
| IFNG | 6.62 | 9.37 | 0.10 |
| IL1B | 6.90 | 1.86 | 0.12 |
| IL2 | 323.83 | 758.20 | 0.01 |
| IL4 | 0.67 | 0.21 | 0.06 |
| IL5 | 0.43 | 0.32 | 0.38 |
| IL6 | 57.13 | 12.62 | 0.18 |
| CXCL8 | 3216.50 | 2969.48 | 0.30 |
| IL9 | 1.10 | ↓ SC | n/a |
| IL10 | 5.15 | 1.80 | 0.02 |
| IL12A | 1.24 | ↓ SC | n/a |
| IL13 | 5.34 | ↓ SC | n/a |
| IL17A | 0.47 | ↓ SC | n/a |
| CCL2 | 377.78 | 93.76 | 0.04 |
| CCL3 | 7.48 | 13.33 | 0.24 |
| CCL4 | 65.34 | 87.89 | 0.24 |
| TNF | 10.28 | 10.98 | 0.44 |

B: Transcriptional response of different cytokine levels to SARS-CoV-2 infection in lung biopsies (n=2) compared to their healthy counterparts (GEO accession: GSE147507).

| Gene_Symbol | healthy | COVID-19 |
|---|---|---|
| CSF2 | 1017.2458 | 171.7929 |
| IL2 | 3.036295 | 1 |
| IL4 | 2.431805 | 1 |
| IL5 | 3.420165 | 1 |
| IL6 | 25225.35 | 19153.55 |
| IL1B | 5637.209 | 71878.2 |
| IL9 | 1 | 1 |
| IL10 | 121.2407 | 342.545 |
| IL12A | 57.1823 | 1 |
| IL13 | 10.06294 | 1 |
| IL17A | 3.733445 | 1 |
| IFNG | 12.45225 | 1 |
| CCL2 | 18723.99 | 182502 |
| CCL3 | 1736.7923 | 97884.55 |
| CCL4 | 707.44 | 238525.5 |
| TNF | 75.6832 | 2916.045 |

Supplementary Figure 1: The top 20 enriched GO terms from downregulated genes of ACE2$^{high}$ vs. ACE2$^{low}$ MCF-7 cells.

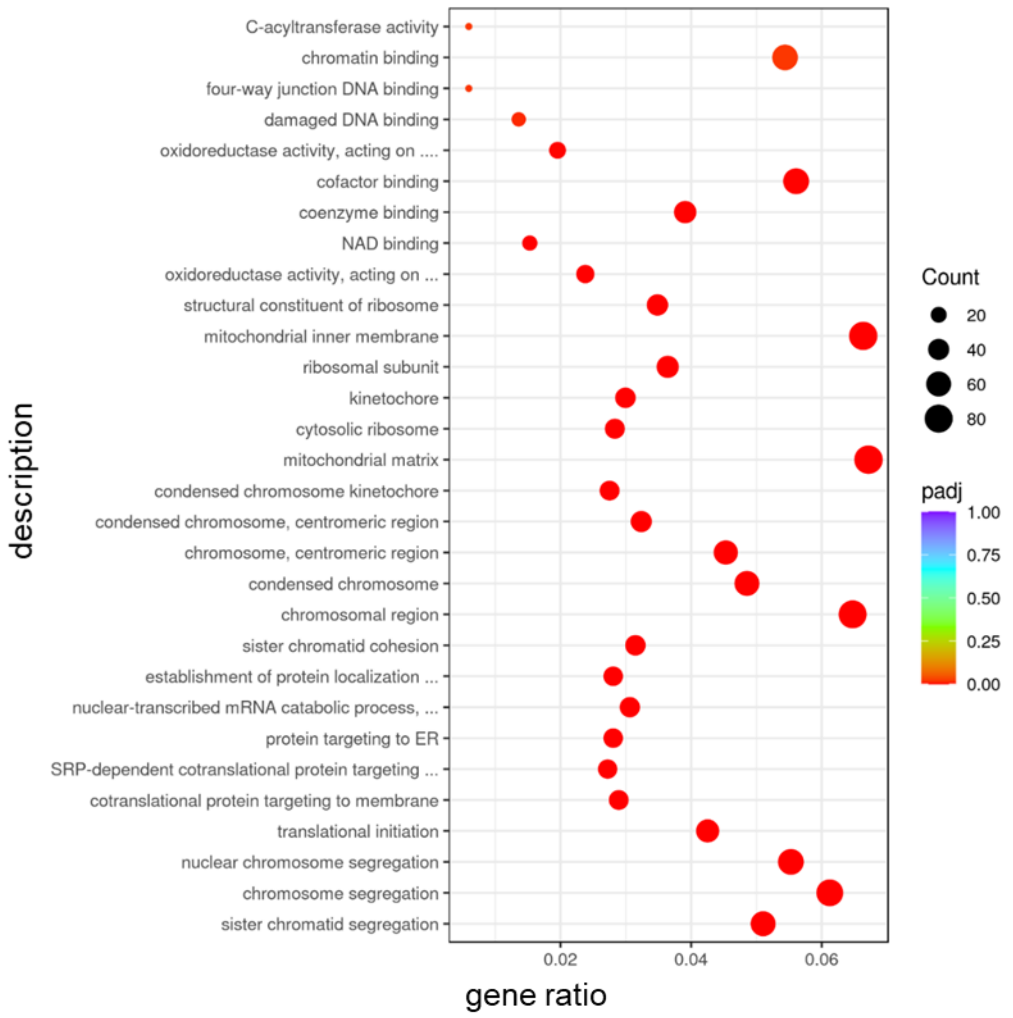

Supplementary Figure 2: Common disease annotations pattern of DEGs of ACE2$^{high}$ vs. ACE2$^{low}$ MCF-7 cells.

A: Disease annotations of the top 5 upregulated genes demonstrated a link to viral infections, most commonly to one disease term, influenza (disease id: C0021400)

B: the downregulated genes were annotated to two disease terms of BC (malignant tumor of breast (disease id: C0006142) and breast carcinoma (disease id: C0678222). The Venn diagram was made using InteractiVenn.

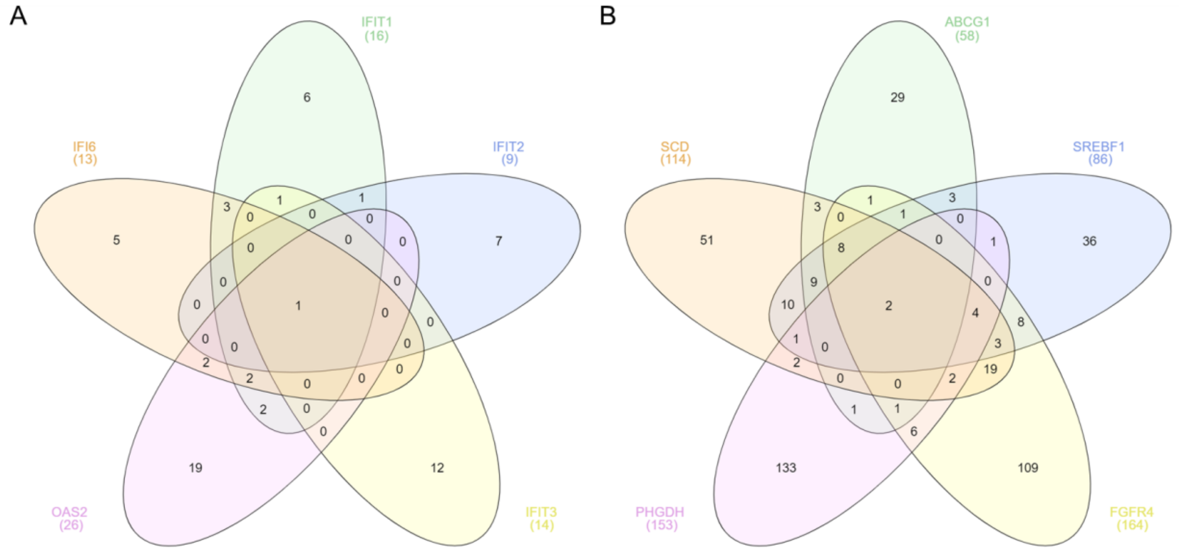


Supplementary Figure 3: Commonly enriched upregulated genes of DEGs of ACE2$^{high}$ vs. ACE2$^{low}$ MCF-7 cells. A Venn diagram was made to the top five GO terms, namely 'defense response to other organism', 'response to type I IFN', 'defense response to virus', 'response to virus', and 'type I IFN signaling pathway'. The 14 genes include OAS1, OAS2, OAS3, OASL, STAT1, IFITM3, IRF1, IRF2, IRF7, IRF9, BST2, IFITM1, IFITM2 and NLRC5.

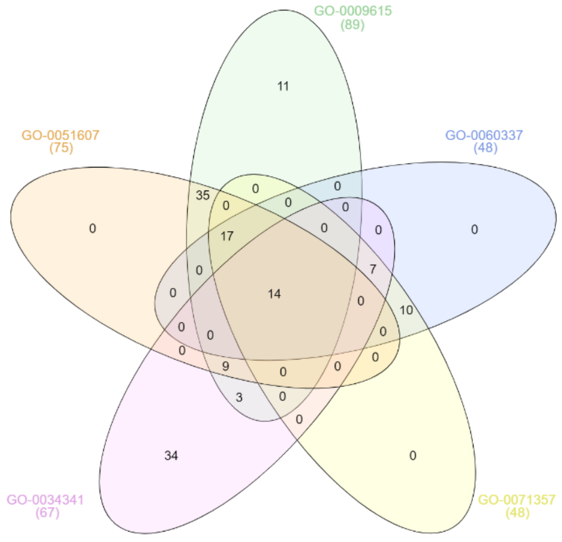

Supplementary Figure 4: Comparison of the upregulated gene profile of ACE2$^{high}$ cells with the SARS-CoV-2 and other respiratory viruses infected cells (GEO accession: GSE147507).
A, B: top 10 up- and downregulated genes of ACE2$^{high}$ MCF-7 cells with the SARS-CoV-2 infections (A) and other respiratory viruses such as IAV, IAVdNS1, HPIV3 and RSV (B).

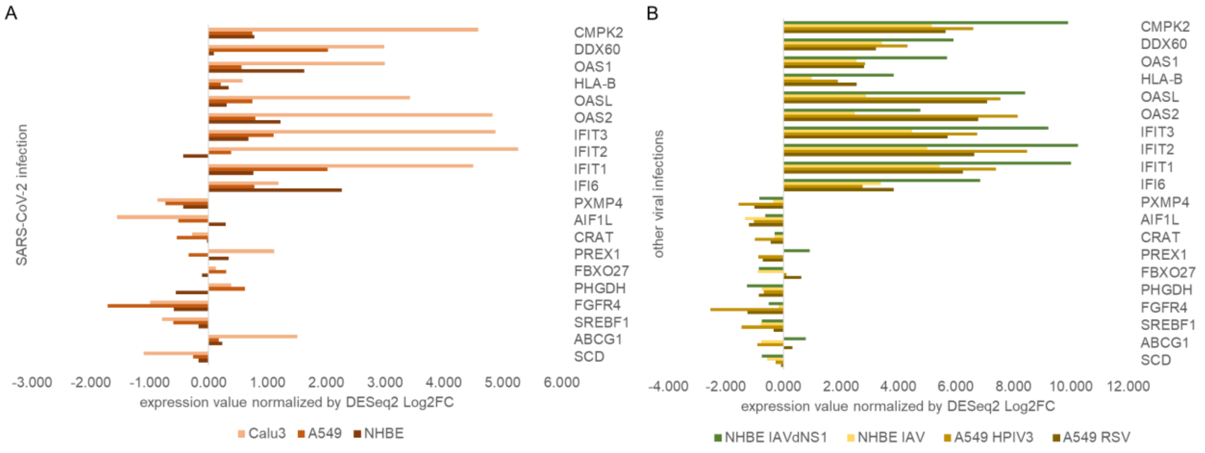


Supplementary Figure 5: Transcriptional response of HLA-A, -B and –C and PD-L1 levels to SARS-CoV-2 infection in lung biopsies (n=2) compared to their healthy counterparts (GEO accession: GSE147507).

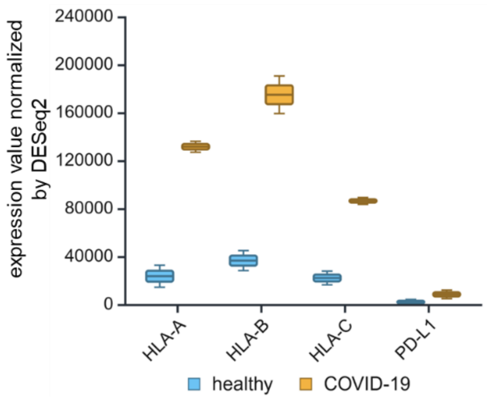


Supplementary Figure 6: Transcriptomic expressions of HLA-A, -B and -C in cell lines after infection with other respiratory viruses, such as IAV and IAVdNS1 on cell line NHBE, HPIV3 and RSV on cell line A549 (GEO accession: GSE147507).

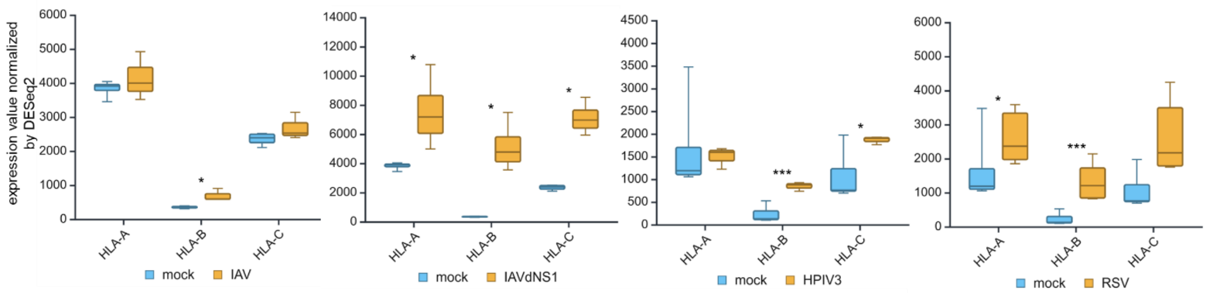

Supplement: Supplementary file 1 — Supplementary Material 1 [file 18_2024_5520_MOESM1_ESM.pdf]
